# Adverse Effects of fine particulate matter on human kidney functioning: a systematic review

**DOI:** 10.1186/s12940-021-00827-7

**Published:** 2022-02-08

**Authors:** Leen Rasking, Kenneth Vanbrabant, Hannelore Bové, Michelle Plusquin, Katrien De Vusser, Harry A. Roels, Tim S. Nawrot

**Affiliations:** 1 Centre for Environmental Sciences, Hasselt University, Agoralaan Gebouw D, B-3590 Diepenbeek, Belgium; 2 Nephrology and Kidney Transplantation, University Hospital Leuven, Leuven, Belgium; 3 Department of Microbiology, Immunology, and Transplantation, Leuven University, Leuven, Belgium; 4 Louvain Centre for Toxicology and Applied Pharmacology, Université catholique de Louvain, Brussels, Belgium; 5 Department of Public Health and Primary Care, Environment and Health Unit, Leuven University, Leuven, Belgium

**Keywords:** Air pollution, Fine particulate matter, PM_2.5_, Kidney, Kidney disease

## Abstract

**Background:**

Ambient fine particulate matter (PM < 2.5 μm, PM_2.5_) is gaining increasing attention as an environmental risk factor for health. The kidneys are considered a particularly vulnerable target to the toxic effects that PM_2.5_ exerts. Alteration of kidney function may lead to a disrupted homeostasis, affecting disparate tissues in the body. This review intends to summarize all relevant knowledge published between January 2000 and December 2021 on the effects of ambient PM_2.5_ and the adverse effects on kidney function in adults (≥ 18 years).

**Results and Discussion:**

Studies published in peer-reviewed journals, written in English, regarding the effects of PM_2.5_ on kidney function and the development and/or exacerbation of kidney disease(s) were included. Of the 587 nonduplicate studies evaluated, 40 were included, comprising of studies on healthy or diagnosed with pre-existing disease (sub)populations. Most of the studies were cohort studies (n = 27), followed by 10 cross-sectional, 1 ecological and 2 time-series studies. One longitudinal study was considered intermediate risk of bias, the other included studies were considered low risk of bias. A large portion of the studies (n = 36) showed that PM_2.5_ exposure worsened kidney outcome(s) investigated; however, some studies show contradictory results. Measurement of the estimated glomerular filtration rate, for instance, was found to be positively associated (n = 8) as well as negatively associated (n = 4) with PM_2.5_.

**Limitations and Conclusion:**

The main limitations of the included studies include residual confounding (e.g., smoking) and lack of individual exposure levels. The majority of included studies focused on specific subpopulations, which may limit generalizability. Evidence of the detrimental effects that ambient PM_2.5_ may exert on kidney function is emerging. However, further investigations are required to determine how and to what extent air pollution, specifically PM_2.5_, exerts adverse effects on the kidney and alters its function.

**Registration:**

The systematic review protocol was submitted and published by the International Prospective Register of Systematic Reviews (PROSPERO; CRD42020175615).

**Supplementary Information:**

The online version contains supplementary material available at 10.1186/s12940-021-00827-7.

## Background

The human kidneys are a vulnerable target for exposure to toxic substances due to their filtration function. About 180 L of blood are filtered per day, of which water, metabolic waste, and toxic components are removed [1]. Altered kidney function may affect homeostasis and, subsequently, lead to dysfunctions in other tissues [2, 3]. Kidney diseases, such as chronic kidney disease (CKD), hold a large burden on public health worldwide [4]; it is estimated that globally, e.g., CKD prevalence amounts to 13.4% (11.7 – 15.1%) and progression of the disease may lead to end-stage renal disease (ESRD), requiring dialysis and/or kidney replacement therapy [5]. Furthermore, significant costs can be attributed to requiring dialysis; in 2018, the annual cost for dialysis per person was estimated at 85,966 USD (76,282 EUR) [6].

A significant toxic substance to which everyone is exposed on a daily basis is particulate matter (PM) from ambient air pollution. PM is classified by the International Agency for Research on Cancer (IARC) as carcinogenic to humans [7]. Rather than coarse PM (PM_10_) as the indicator of airborne particulate pollution, fine particulate matter (PM_2.5_; particles having a diameter < 2.5 microns) has been gaining more attention and is assumed to be more closely associated with adverse health effects linked to outdoor air pollution exposure [8]. In 2016, the World Health Organization (WHO) estimated that annually 4.2 million deaths could be attributed to ambient PM_2.5 _ [9]. As of now, no threshold for PM_2.5_ has been identified below which it would not pose a threat to human health. Therefore, in 2021, the WHO lowered the annual mean of PM_2.5_ from 10 µg/m³ to 5 µg/m³ [10]. PM_2.5_ has the potential to translocate into the blood [11] and towards distant organs [12, 13] following inhalation. In that regard, Saenen et al. [14] showed the presence of black carbon particles – a significant component of PM_2.5_ – as a marker of medium-term to chronic exposure to combustion-related air pollution in the urine of healthy individuals. The presence of these toxic particles may cause direct or indirect adverse effects on the kidneys. In that regard, PM_2.5_ has been shown to mediate atherosclerosis development, which may induce vascular dysfunction and result in microvascular damage and atherosclerotic kidney disease [15]. This suggests that inhaled small particles (<30 nm diameter) can selectively accumulate in the kidney during the filtration and excretion processes and subsequently directly induce vascular inflammation, entailing renal damage. Moreover, persons already diagnosed with a disease affecting the kidney (e.g., diabetes mellitus) or with a compromised immune system (e.g., kidney transplant recipients) could experience a worsening of symptoms following increased PM_2.5_ exposure [16].

The detrimental effects of air pollutants such as PM_2.5_ on the kidney have just begun to be acknowledged. Therefore, this systematic review aims to (i) summarize the literature regarding ambient PM_2.5_ exposure and its adverse effects on kidney functioning in humans, (ii) to elucidate the reported detrimental effects on the kidneys, and (iii) to evaluate the research gaps and further research needs.

## Methods

This systematic review was processed according to the Preferred Reporting Items for Systematic reviews and Meta-Analyses (PRISMA) statement [17]. In accordance with these guidelines, our systematic review protocol was submitted and published by the International Prospective Register of Systematic Reviews (PROSPERO; CRD42020175615).

### Data Searches and Sources

Studies addressing the potential effects of ambient PM_2.5_ exposure on kidney functioning in adults (≥ 18 years) were retrieved according to a four-stage process. In the first stage, potentially eligible studies were identified through a literature search of two bibliographic databases, PubMed (www.pubmed.ncbi.nlm.nih.gov) and Scopus (www.elsevier.com/solutions/scopus), using the MeSH terms “kidney” and “kidney disease” along with the keywords: “fine particulate matter”, “element* carbon”, “black carbon”, “ufp”, ultrafine partic*”, particul* matter”, “PM_2.5_”, and “nephropath*”, “kidney failure”, “kidney insufficienc*”, “renal insufficienc*”, and “chronic renal”. The reference lists of key reviews and the included papers were screened to recover any additional eligible publications to ensure literature completeness. The literature search covered articles published between January 1st, 2000 and December 20th, 2021.

### Data Selection and Risk of Bias Evaluation

In the second stage, two reviewers (LR and KVB) independently screened the titles and abstracts of all identified papers to exclude studies that did not fulfill one or more of the *a priori* set inclusion criteria. Any disagreement was resolved through discussion. If no consensus could be reached, a third reviewer (HB) was consulted. Duplicate studies were removed. We included longitudinal, cross-sectional, and cohort studies written in English, which addressed the effects of exposure to PM_2.5_ on the kidney or kidney disease outcomes. Publications describing animal or *in vitro* studies, or examining exposure to coarse PM (PM_10_), PM_2.5 − 10_, and volatile substances [e.g., carbon monoxide (CO), ozone (O_3_), nitrogen dioxide (NO_2_), or sulfur dioxide (SO_2_)] were excluded. Additionally, studies focusing on kidney function or disease outcomes in children or adolescents were excluded as the leading causes of the development of kidney diseases vary significantly among these subpopulations [18, 19].

In a third stage, full-text articles were retrieved and underwent a second screening for eligibility following the previously described inclusion criteria. According to the Newcastle-Ottowa Quality Assessment Scale (NOS) for cohort studies, risk of bias analysis was performed by two independent researchers (LR, KVB). The NOS scale uses a star system to judge a study and to evaluate the risk of bias [20]. The most important adjustable factor for comparability was considered age. The cut-off for the highest risk of bias was set at less than half of the points obtainable (3 stars or less).

### Data Extraction

In the fourth stage, selected studies were grouped according to the specified kidney disease outcome(s) under investigation characterizing the study population. To conduct the in-depth systematic review, the following information was extracted and registered from each article in a preset data extraction form: authors, publication year, country where the study is realized, study period, study population, type of study, PM_2.5_ exposure measurements, kidney dysfunction or parameters investigated, comorbidity at onset of the study (e.g., diabetes mellitus), identified confounders, and main findings (e.g., incidence rate of kidney disease outcome(s) in relation to PM_2.5_ exposure).

### Synthesis of Results

The diversity in the examined human populations (e.g., the elderly, pregnant women, or general adult population) and differently defined assessments of kidney disease outcomes (e.g., using only one, two, or more eGFR measurements to determine CKD) did not allow to carry out a comparative quantitative analysis. Alternatively, we provided a qualitative overview of the results describing the effects of PM_2.5_ exposure on human kidney disease outcomes. The narrative synthesis of results was subsequently achieved by summarizing and grouping information on different kidney disease outcomes in relation to PM_2.5_ exposure.

## Results

### Literature Selection and Assessment of Risk of Bias

**Fig. 1:**
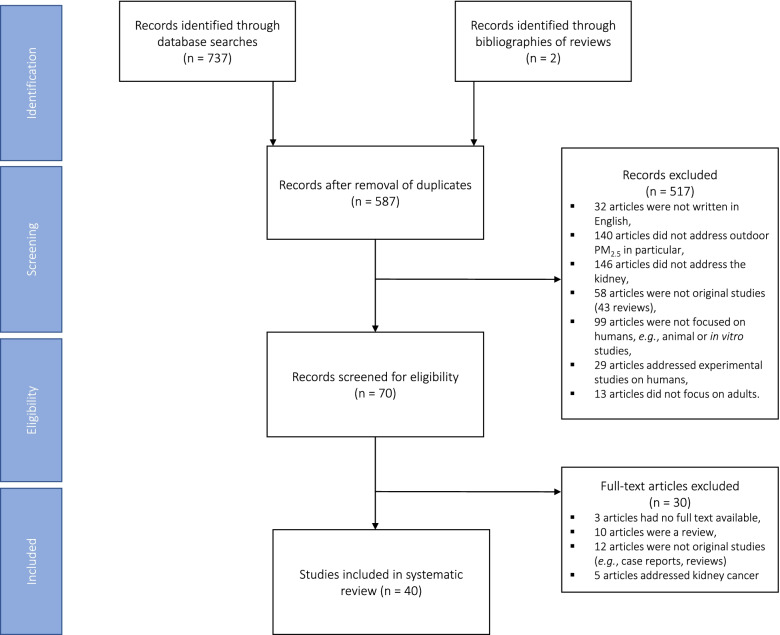
Overview of the data selection process. Records either identified through database searches of PubMed and Scopus (n = 737) from January 1st, 2000 until December 20th, 2021, and records identified through bibliographies of reviews (n = 2). After checking for and removal of duplicates, records were screened for eligibility according to the a priori defined criteria, that resulted in n = 70 eligible records. Next, full-text articles were screened for a second time against the eligibility criteria. The final selection (n = 40) consisted of human studies focusing on ambient PM exposure and addressing: (i) clinical measurements of eGFR (n = 8), (ii) general kidney function through changes in biomarkers (n = 2), and (iii) glomerular diseases (n = 4). The remaining articles focused on persons with a diagnosis of diabetes mellitus where kidney function declined (n = 2), CKD (n = 14), ESRD (n = 2), renal failure (n = 4), and kidney transplant outcome (n = 4). Abbreviations: CKD, chronic kidney disease; ESRD, end-stage renal disease; eGFR, estimated glomerular filtration rate; PM_2.5_, fine particulate matter (< 2.5 microns)

The last search was conducted on December 20^th^, 2021 using the MeSH terms “kidney” and “kidney disease”, and the aforementioned additional keywords to identify 737 articles in total. Also, two new articles were identified from reference lists of reviews (Fig. 1). After the removal of duplicates, 587 articles remained and were screened for eligibility. The abstracts of these 587articles were evaluated and 517 records were excluded from the analysis. From the total of excluded records, 32 articles were not written in English, 140 articles did not focus on ambient PM_2.5_specifically, and 146 did not address the kidney or kidney disease outcomes. Additionally, 199 articles were excluded from the analysis as they did not address original studies (n = 58), focused on experimental studies in animals or humans (n = 99+29 = 128), or did not address adults (n = 13). The remaining 70 selected studies underwent in-depth review, resulting in 30 additional removals (3 articles lacked full-text availability, 10 were reviews, 12 were not original reports, and 5 addressed kidney cancer). The 40 studies fulfilling the inclusion criteria were included for this systematic review.

The risk of bias analysis was performed using the Newcastle – Ottowa Scale and articles were ranked accordingly: gaining ≤ 3 stars meant a high risk of bias, ≤ 5 stars an intermediate risk of bias, and ≥ 6 stars a low risk of bias [20]. One article had an intermediate risk of bias [21], while no articles were considered as high risk of bias (Supplementary Table 1).

### Study Characteristics

**Fig. 2:**
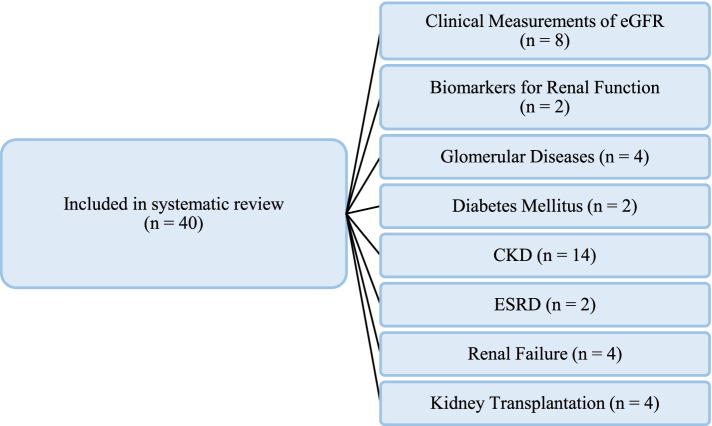
Schematic overview of the distribution of articles on kidney disease (outcomes) in the systematic review. Eligible articles (n = 40) were divided into the corresponding disease outcomes. Included studies focused on (i) clinical measurements of eGFR (n = 8), (ii) biomarkers to evaluate kidney function following PM exposure (n = 2), (iii) glomerular diseases (n = 4), (iv) diabetes mellitus as a driver to kidney function decline (n = 2), (v) CKD (n = 14), (vi) ESRD (n = 2), (vii) kidney failure (n = 4), and (viii) kidney transplantation (n = 4). These numbers of articles for the listed classes of kidney disease (outcomes) do not necessarily correspond with the main text, as for each class, the most representative articles were considered. Abbreviations: CKD, chronic kidney disease; ESRD, end-stage renal disease; eGFR, estimated glomerular filtration rate; PM_2.5_, fine particulate matter (< 2.5 microns)

The 40 studies included in this systematic review were either cohort studies (n = 27, Table 1), cross-sectional (n = 10, Table 2), ecological (n = 1, Table 3), or a time-series study (n = 2, Table 3) and conducted in an epidemiological context of exposure to ambient PM_2.5_. We identified studies investigating (i) clinical measurements of the estimated glomerular filtration rate (eGFR) (n = 8) [15, 21–27], (ii) general kidney functioning through changes in biomarkers (n = 2) [28, 29], and (iii) glomerular diseases (n = 4) [30–32], including systemic lupus erythematosus (SLE; n = 1) [33]. The remaining 26 articles focused on persons with a diagnosis of diabetes mellitus (n = 2) [34, 35], the development and/or worsening of CKD (n = 14) [36–49], ESRD (n = 2) [50, 51], renal failure (n = 4) [52–55], and kidney transplant outcome (n = 4) (Fig. 2) [56–59].

**Table 1:** Data extraction of the 27 studies in the systematic review with a cohort study design

Authors (year) (type of study)	Exposure Estimate	Study Population	Main Findings
**Fang** et al. **(2020) ** [21] (panel study)	The mean ± SD 72-hour PM_2.5_ concentration was 54.57 ± 46.21 µg/m³, with an IQR of 31.43 µg/m³. The average PM_2.5_ concentration far exceeded the WHO air quality guidelines.	Chinese persons residing in Jinan (n = 71) aged between 60 and 69 years with a mean ± SD age of 65.1 ± 2.8 years. Study period: Sept. 2018 – Jan. 2019	An IQR increment of total PM_2.5_ concentration was significantly associated with a 3.27% reduction in eGFR (p < 0.05) for the lag period of 0 – 24 h.
**Blum** et al. **(2020) **[22] (prospective cohort study)	The median annual average ± SD PM_2.5_ concentrations were 15.3 ± 1.0 µg/m³, 12.2 ± 0.7 µg/m³, 9.4 ± 0.8 µg/m³, and 14.6 ± 1.2 µg/m³ for Forsyth County, Jackson, Minneapolis, and Washington County, respectively. The average PM_2.5_ concentration exceeded the newly set WHO air quality guidelines for all counties.	Participants from the Atherosclerosis Risk in Communities cohort (n = 10,997). Mean ± SD age at the last visit was 63 ± 6 years. Study period: 1996 – 2016	No significant association between PM_2.5_ exposure and eGFR could be shown at baseline. A higher annual average PM_2.5_ exposure was associated with increased albuminuria (p ≤ 0.001) and a higher risk of developing CKD (p < 0.05).
**Mehta** et al. **(2016) **[23] (prospective cohort study)	The mean average ± SD 1-year PM_2.5_ exposure levels were 11.4 ± 1.0 µg/m³ at the first visit and 10.5 ± 1.4 µg/m³ across all visits. The average PM_2.5_ concentration exceeded the WHO air quality guidelines.	Participants from the Veterans Administration Normative Aging Study (n = 669) with a mean ± SD age of 73.5 ± 6.8 years. Study period: 2000 – 2011	One-year PM_2.5_ exposure was significantly (p < 0.05) associated with reduction in eGFR and an additional annual decrease in eGFR.
**Li A.** et al. **(2021) **[24] (prospective cohort study)	For PM_2.5_, the 7-day moving average concentrations were 84.8 ± 38.9, 55.5 ± 29.6, and 40.1 ± 20.5 µg/m³ at the first through third visit, respectively. These concentrations far exceeded the air quality guidelines set by the WHO.	Participants residing in Beijing, China (n = 169) with an average ± SD age of 64.0 ± 8.7 years. Study period: Nov. 2016 – 2018	No associations could be found between PM_2.5_ exposure and eGFR or UACR (p > 0.05).
**Feng Y.M.** et al. **(2021)** [25] (prospective cohort study)	The median PM_2.5_ level was 13.1 µg/m³ (5th to 95th percentile interval, 12.4 to 15.3 µg/m³). The levels exceeded the air quality guidelines set by the WHO.	Flemish residents (Belgium) (n = 820 at baseline and n = 653 at follow-up) with a mean follow-up of 4.7 years. Study period: 2005 – 2009	No renal outcome (eGFR, serum creatinine, microalbuminuria, and CKD) could be associated to PM_2.5_ exposure levels when observing only the baseline participation, only the follow-up participation, or a combination (p > 0.05).
**Li Q.** et al. **(2021)** [26] (prospective cohort study)	The median PM_2.5_ exposure was 61.0 µg/m³ (IQR: 49.0 to 75.5 µg/m³) of all participants. The mean ± SD PM_2.5_ exposure was 60.9 ± 15.7 µg/m³. The PM_2.5_ levels exceeded by far the air quality guidelines set by the WHO.	Chinese residents of Han ethnicity (n = 1,280,750 females and n = 1,256,297 males) who were ≥ 18 to ≤ 45 years of age. Study period: Jan. 2013 – Oct. 2014	Significant differences in serum creatinine and eGFR could be observed for each 10 µg/m³ increment of PM_2.5_ exposure. The association was higher in females compared to males (p < 0.05).
**Gao** et al. **(2021) **[27] (prospective cohort study)	The average mean ± SD 28-day PM_2.5_ levels were 9.27 ± 3.08 µg/m³. The average PM_2.5_ concentration exceeded the WHO air quality guidelines.	Participants form the Veterans Administration Normative Aging Study (n = 808; study visits = 2,466) with a mean ± SD age of 75.7 ± 7.2 years. Study period: 1998 – 2016	Short-term (28-day) exposure to ambient PM_2.5_ could be robustly associated to a decrease in eGFR (p < 0.001), but could not be associated to serum uric acid, blood urea nitrogen, and odds of CKD (p ≥ 0.06).
**Xu** et al. **(2016)** [30] (cohort study)	The 3-year average PM_2.5_ exposure varied, ranging from 6 – 114 µg/m³ with a mean of 52.6 µg/m³. The average PM_2.5_ concentration far exceeded the WHO air quality guidelines.	Patients providing a renal biopsy in 938 hospitals spanning 282 cities in China (n = 71,151). Of the total participants, the mean ± SD age was 37.3 ± 15.9 years. Study period: 2004 – 2014	A 10 µg/m³ increment in PM_2.5_ exposure was associated with 14% higher odds for membranous nephropathy at PM_2.5_ > 70 µg/m³; no association was shown at PM_2.5_ < 70 µg/m³. The annual increase in risk for MN was greater in cities with higher slopes of PM_2.5_ exposure. A higher 3-year average PM_2.5_ concentration was associated with an increased risk of membranous nephropathy.
**Lin S.Y.** et al. **(2018)** [31] (cohort study)	The daily average ± SD PM_2.5_ amounted to 34.8 ± 8.76 µg/m³. PM_2.5_ exposure levels were divided into 4 quartiles: Q1 (<29.5 µg/m³), Q2 (29.5 – 33.2 µg/m³), Q3 (33.3 – 41.2 µg/m³), and Q4 (>41.2 µg/m³). The average PM_2.5_ concentration far exceeded the air quality guidelines set by WHO.	Persons registered in the Longitudinal Health Insurance Database (n = 161,970) in Taiwan with a mean ± SD age of 40.5 ± 14.6 years. Follow-up time (mean ±SD): 11.7 ± 0.99 years Study period: Jan. 2000 – Dec. 2011	Increasing quartile concentrations of PM_2.5_ were associated with an increased risk of nephrotic syndrome (p ≤ 0.05). Similar results were obtained when stratified by the follow-up period (≤ 6 years).
**Bowe** et al. **(2020)** [34] (prospective cohort study)	PM_2.5_ exposure levels were divided into 4 quartiles: Q1 (5.0 – 10.1 µg/m³), Q2 (10.2 – 11.8 µg/m³), Q3 (11.9 – 13.7 µg/m³), and Q4 (13.8 – 22.1 µg/m³). The PM_2.5_ concentrations of all quartiles exceeded the new WHO air quality guidelines.	War veterans with diagnosed diabetes mellitus (n = 2,444,157) from the United States with a median (IQR) age of 62.5 (54.7 to 71.8) years. Follow-up time (median): 8.5 years Study period: Oct. 2003 – Sept. 2012	Adjusted incidence rates of kidney disease outcomes were elevated across increasing PM_2.5_ quartiles. A 10 µg/m³ increment in PM_2.5_ was individually associated with increased odds of diabetes and increased risk of kidney disease outcomes. Diabetes may be a mediator in the relationship between PM_2.5_ exposure and kidney disease outcomes.
**Chin** et al. **(2018)** [35] (cohort study, longitudinal analysis)	The mean ± SD PM_2.5_ exposure level was 34.1 ± 6.0 µg/m³. PM_2.5_ exposure levels were subdivided into quartiles: Q1 (27.7 µg/m³), Q2 (data not shown), Q3 (38.8 µg/m³), and Q4 (data not shown). The average PM_2.5_ concentrations far exceeded the WHO air quality guidelines.	Patients diagnosed with diabetes mellitus type II (n = 812) from Taiwan with a mean ± SD age of 55.4 ± 8.4 years. Study period: 2003 – 2012	The annual increase of ACR was positively associated with PM_2.5_ exposure (p < 0.05). A more rapid progression of microalbuminuria was seen in patients exposed to higher levels of PM_2.5_.
**Chan** et al. **(2018) **[37] (cohort study, longitudinal analysis)	The overall average mean ± SD for PM_2.5_ exposure was 27.1 ± 8.0 µg/m³ with an IQR of 10.4 µg/m³, exceeding the air quality guidelines set by WHO. Baseline PM_2.5_ exposure increased slightly from 2001 to 2004 and then declined, but remained relatively stable from 2005 to 2011.	General Taiwanese adult population with a mean ± SD age of 38.9 ± 11.3 years (n = 100,629). Of the participants, 4,046 incident CKD cases developed during the follow-up period of 10 years. Study period: 1994 – 2014	Higher levels of PM_2.5_ exposure was associated with a higher risk of developing CKD (p < 0.05). A significant dose-response trend was observed, with a 6% increased risk of developing CKD for a 10 µg/m³ increment of PM_2.5_ (p < 0.05).
**Lin S.Y.** et al. **(2020)** [39] (prospective nation-wide cohort study)	The inverse distance weighing method was used to calculate annual average PM_2.5_ exposure and to estimate the annual exposure for each patient (average ± SD: 34.8 ± 8.76 µg/m³). PM_2.5_ exposure was divided into 4 quartiles: Q1 (<29.5 µg/m³), Q2 (29.5 – 33.3 µg/m³), Q3 (33.3 – 41.2 µ/m³), and Q4 (≥41.2 µg/m³). An IQR value was set at 8.3 µg/m³ PM_2.5_. The average PM_2.5_ concentration exceeded the air quality guidelines set by WHO.	Adult participants with a mean ± SD age of 40.3 ± 14.5 years residing in Taiwan (n = 161,970). Median (IQR) follow-up time: 11.9 (11.8 – 12) years Study period: 1998 – 2011	A higher risk of CKD was associated with increasing levels of PM_2.5_ exposure (p < 0.001). The risk of ESRD development was increased with PM_2.5_ exposure in a similar trend as the increased risk of developing CKD (p ≤ 0.01).
**Ran** et al. **(2020a)** [40] (prospective cohort study)	The annual mean ± SD concentration of PM_2.5_ exposure level was 37.8 ± 2.9 µg/m³ with an IQR of 4.0 µg/m³ at the baseline of the study. The average PM_2.5_ concentration exceeded the WHO air quality guidelines by almost four-fold.	Adults > 65 years from the Hong Kong Elderly Health Service cohort (n = 66,820) of whom 902 participants developed CKD (mean ± SD age: 72.8 ± 6.0 years). Study period: 1998 – 2010	PM_2.5_ exposure was associated with the hazard of developing CKD in the presence of hypertension. A higher risk of all-cause mortality was associated with PM_2.5_ exposure. An increased risk for renal failure and mortality risk of renal failure was shown in association with an IQR increment of PM_2.5_; the latter for CKD patients with existing hypertension. Furthermore, concentration-response relationships of all-cause and renal failure mortality risks associated with PM_2.5_ were demonstrated.
**Jung** et al. **(2021)** [43] (retrospective cohort study)	The mean PM_2.5_ levels were 24.84 and 24.37 µg/m³ for CKD patients who died and survived during follow-up, respectively. Both mean values exceeded the air quality guidelines set by the WHO.	A subset of the South Korean population (n = 18,717) consisted of CKD patients (whom had PM_2.5_ exposure data available) with a mean ± SD age of 57 ± 17 years with a follow up of mean ± SD of 4.10 ± 2.51 years. Study period: 2001 – 2015	A significant effect was observed between PM_2.5_ levels and mortality in CKD patients (p = 0.019). Long-term exposure was shown to have negative effects on mortality in CKD patients.
**Ghazi** et al. **(2021)** [44] (cohort study)	The median PM_2.5_ concentration was 10.1 µg/m³ for the overall cohort. At baseline, PM_2.5_ levels were <9.5 µg/m³, 9.5 to 10.1 µg/m³, 10.1 to 10.7 µg/m³, and ≥10.7 µg/m³ for Q1, Q2, Q3, and Q4, respectively.	Adult patients (≥18 years old; n = 113,725) with an average ± SD age of 50 ± 18 years (Minnesota, USA). Study period: Jan. 2012 – Apr. 2019	11% of the population had CKD. Increased risk and greater odds for developing CKD was observed for patients who had elevated levels of PM_2.5_ exposure (p < 0.05).
**Bo** et al. **(2021)** [46] (cohort study)	The 2-year average ± SD PM_2.5_ levels amounted to 26.7 ± 7.7 µg/m³. These levels exceed the air quality guidelines set by the WHO.	Taiwanese residents (n = 163,197) with a mean ± SD age of 38.4 ± 11.6 years at recruitment. The average follow-up period was 5.1 years (range from 1.0 to 7.4 years). Study period: 1996 – 2016	A linear concentration-response relationship was shown between average PM_2.5_ levels and incidence of CKD. Each 5 µg/m³ decrease in ambient PM_2.5_ concentration could be associated with a reduced risk of CKD development (p < 0.001).
**Zeng** et al. **(2021)** [47] (longitudinal cohort study)	The mean ± SD concentration of PM_2.5_ amounted to 26.8 ± 7.8 to 7.9 µg/m³ (SD for incidence of eGFR decline ≥30% and CKD incidence, respectively). The air quality guidelines set by the WHO were exceeded.	Taiwanese participants (total of n = 108,615 for eGFR and n = 104,092 for CKD analysis) were included to investigate the effect on incidence of eGFR decline ≥30% and CKD incidence, with a mean ± SD follow-up period of 6.7 ± 3.2 years. Study period: 2001 – 2016	A moderate to high exposure to PM_2.5_ was associated with a higher risk of incident eGFR decline ≥30% and incident CKD (p < 0.001). Associations were also positive per 10 µg/m³ increment of PM_2.5_ (p < 0.001).
**Wu** et al. **(2020)** [50] (prospective cohort study)	PM_2.5_ exposure was divided into 4 quartiles: Q1 (11.71 – 28.69 µg/m³), Q2 (28.69 – 30.16 µg/m³), Q3 (30.16 – 39.96 µ/m³), and Q4 (39.96 – 46.63 µg/m³), with all quartiles exceeding the WHO air quality guidelines. An IQR value was set at 11.31 µg/m³.	Adults registered in the National Health Insurance Research Database from Taiwan (n = 623,894). Of the participants, 1,945 subjects developed ESRD during the study period. Study period: 2003 – 2012	A significant positive association was found between PM_2.5_ exposure and incidence of ESRD (p < 0.05). Participants in the highest quartile of exposure to PM_2.5_ had a significantly higher risk of developing ESRD and a higher cumulative incidence of ESRD compared to participants in the 1st quartile (p < 0.05).
**Bowe** et al. **(2018)** [51] (prospective cohort study)	PM_2.5_ exposure was divided into 4 quartiles: Q1 (5.0 – 9.1 µg/m³), Q2 (9.2 – 11.0 µg/m³), Q3 (11.1 – 12.6 µ/m³), and Q4 (12.7 – 22.1 µg/m³). Two of the quartiles had average PM_2.5_ concentrations that exceeded the WHO air quality guidelines.	War veterans (USA) with a median age (IQR) of 62.46 (54.68 – 71.78) years (n = 2,482,737) with a median follow-up period of 8.52 years. Study period: Oct. 2003 – Sept. 2012	An increased risk of incident eGFR <60mL/min/1.73 m², an eGFR decline ≥30%, incident CKD, and an increased risk of developing ESRD was shown for 10 µg/m³ increment in PM_2.5_ exposure (p ≤ 0.05). A linear relationship was observed between PM_2.5_ exposure and risk of eGFR decline ≥ 30%.
**Ran** et al. **(2020b)[**52] (retrospective cohort study)	Median value for PM_2.5_ exposure was 35.78 µg/m³ at the baseline study period (1998 – 2000). An IQR of 3.22 µg/m³ PM_2.5_ was identified. The median PM_2.5_ concentration far exceeded the WHO air quality guidelines.	Elderly population (Hong Kong) with a mean ± SD age of participants of 72.0 ± 5.6 years (n = 61,447). Study period: 1998 – 2010	PM_2.5_ exposure was associated with a higher risk of renal failure mortality in the entire cohort (p < 0.01) and in the subgroup analysis of incident CKD (p ≤ 0.01). An IQR increment of PM_2.5_ led to elevated mortality risk of AKI, but not CKD or unspecified renal failure.
**Lin Y.T.** et al. **(2020)** [53] (prospective cohort study)	PM_2.5_ exposure was divided into 4 quartiles: Q1 (<32.08 µg/m³), Q2 (32.08 – 36.27 µg/m³), Q3 (36.27 – 39.88 µ/m³), and Q4 (≥39.88 µg/m³). An IQR value was set at 7.8 µg/m³. All of the quartiles’ PM_2.5_ concentrations exceeded the WHO air quality guidelines.	Adult Taiwanese participants between the age of 20 – 90 years with a mean (IQR) age of 67.8 (57.5 to 76.6) years and diagnosed with CKD (n = 6,628). Study period: 2003 – 2015	A positive relationship between PM_2.5_ exposure and risk for kidney failure requiring replacement therapy was demonstrated for PM_2.5_ increments of 10 µg/m³ and IQR of 7.8 µg/m³. Furthermore, increased risk of progression to kidney failure requiring replacement therapy was shown across increasing PM_2.5_ quartiles. A significant increasing linear trend in risk for progression to kidney failure across the increasing PM_2.5_ exposure levels was shown (p < 0.001).
**Feng Y.** et al. **(2021a) **[55] (cohort study)	The median PM_2.5_ concentration level amounted to 9.17 µg/m³ (range: 0.70 to 23.62 µg/m³). The levels exceeded the air quality guidelines set by the WHO.	Older kidney failure patients (USA) aged ≥65 years (median age = 74, IQR: 69 to 80 years) at dialysis initiation, who started their first dialysis between 2010 and 2016 (n = 384,276) with a median follow-up of 1.84 years (IQR: 0.77 to 3.25 years). Study period: Jan. 2010 – Dec. 2016	No association could be observed between PM_2.5_ <12 µg/m³ and mortality risk; however, when PM_2.5_ concentrations were >12 µg/m³, associations could be observed with each 10 µg/m³ PM_2.5_ increase in mortality risk among older dialysis patients (p < 0.05). The association appeared nonlinear; the dose-response association changed when the PM_2.5_ levels reached ~12 µg/m³. Furthermore, when diabetes was the primary cause of kidney failure, a higher PM_2.5_-associated mortality risk was observed (p < 0.05).
**Pierotti** et al. **(2018) [**56**]** (retrospective cohort study)	The average median (IQR) PM_2.5_ exposure level was 10.0 (1.4) µg/m³. The average PM_2.5_ concentration exceeded the new WHO air quality guidelines.	Patients who received a kidney transplant between 2000 and 2008 in Great Britain (n = 11,607) with a mean ± SD age of 43.6 ± 15.9 years at transplantation. Study period: Jan. 2000 – Dec. 2008	Exposure to PM_2.5_ was associated with renal transplant failure in univariate analyses, but not after adjustment for confounders. An increased risk of kidney graft failure was shown for each 5 µg/m³ increase in PM_2.5_ (p = 0.03).
**Chang** et al. **(2021) [**57**]** (retrospective cohort study)	The median (IQR) PM_2.5_ level the year before kidney transplantation was 9.8 (8.3 to 11.9) µg/m³. Exposure was divided into 4 quartiles: Q1 (1.2 – <8.3 µg/m³), Q2 (8.3 - <9.8 µg/m³), Q3 (9.8 - <11.9 µg/m³), and Q4 (11.9 - <22.4 µg/m³). The median PM_2.5_ concentration exceeded the newly set air quality guidelines by the WHO.	Patients (USA) receiving a kidney transplant between 2004 and 2016 (n = 112,098) with 62.91% being over 50 years old. Study period: 2004 - 2021	An increased PM_2.5_ level, compared to quartile 1, was not associated with higher odds of acute kidney rejection for quartile 2, but was associated with increased odds for quartile 3 (p < 0.001). Increased PM_2.5_ levels were also associated with an increased risk of death-censored graft failure and all-cause death (p < 0.001)
**Dehom** et al. **(2021) [**58**]** (retrospective cohort study)	The PM_2.5_ concentration levels were divided into 3 tertiles: T1 (2.1 – 9.3 µg/m³), T2 (>9.3 µg/m³ - 11.0 µg/m³), and T3 (>11.0 – 18.4 µg/m³). The medians of all tertiles (T1: 7.9 µg/m³, T2: 10.3 µg/m³, and T3: 11.9 µg/m³) exceeded the air quality guidelines set by the WHO.	Adults (≥18 years; USA) who received a kidney transplant between 2001 and 2015 (n = 93,857) with a median follow-up of 14.91 years. Study period: 2001 – 2015	A 10 µg/m³ increase in PM_2.5_ concentrations was associated with in increased risk of all-cause mortality in kidney transplant recipients (p < 0.05). Black recipients had higher risks of all-cause death than non-blacks. High levels of PM_2.5_ were also associated with all-cause mortality (p < 0.05).
**Feng Y.** et al. **(2021b) [**59**]** (retrospective cohort study)	The median PM_2.5_ level at the time of transplant was 9.2 µg/m³ with a range of 0.7 to 29.7 µg/m³. The median exceeded the new air quality guidelines of the WHO.	Adult kidney transplant recipients (USA) receiving a first transplant between January 1st, 2010, and December 30th, 2016 (n = 87,223) with a median follow-up of 5.3 years. To analyze the results regarding one-year acute rejection, the sample population was restricted to n = 83,669 due to missing follow-up data. Study period: Jan. 2010 – Dec. 2016	A 10 µg/m³ increase in PM_2.5_ concentration was associated with an increased risk of delayed graft function, one-year acute rejection, and all-cause mortality (p < 0.05). When only analyzing the population exposed to PM_2.5_ levels ≤12 µg/m³, no association could be shown with one-year acute rejection. Additionally, no association between an increase of 10 µg/m³ in PM_2.5_ levels and death-censored graft loss.

Air quality guidelines for PM_2.5_ exposure by WHO in 2021 for daily and annual mean are 15 µg/m³ and 5 µg/m³, respectively [10]. The previous guidelines (2006) were 25 µg/m³ and 10 µg/m³, respectively [60]

*Abbreviations*: *(U)ACR* (urinary) albumin-to-creatinine ratio, *AKI* acute kidney injury, *CKD* chronic kidney disease, *eGFR* estimated glomerular filtration rate, *ESRD* end-stage renal disease, *IQR* interquartile range, *PM*_2.5_ fine particulate matter (<2.5 microns), *SD* standard deviation, *WHO* World Health Organization

**Table 2:** Data extraction of the 10 studies in the systematic review with a cross-sectional study design

Authors (year) (type of study)	Exposure Estimate	Study Population	Main Findings
**Zhao** et al. **(2020) [**15**]** (cross-sectional study)	The mean ± SD exposure level to PM_2.5_ during the whole pregnancy was 52.24 ± 2.93 µg/m³ (IQR value: 3.90 µg/m³), exceeding the air quality guidelines set by WHO. The mean ± SD black carbon exposure level during the entire pregnancy was 3.56 ± 0.28 µg/m³.	Healthy pregnant women (n = 10,052) in Shanghai (China) with a mean ± SD gestational age of 35.64 ± 1.74 weeks at renal function testing. Study period: Jan. 2014 – Dec. 2015	An IQR increment in PM_2.5_ was positively associated (p < 0.01) with serum UN in the second and third trimester, and during the whole pregnancy. For serum UA, an IQR increment in PM_2.5_ could only be associated with the first trimester, but not the second or third trimester or during the whole pregnancy. Negative significant associations were demonstrated between eGFR and an IQR increment in PM_2.5_ and black carbon for the first and third trimester of pregnancy as well as the whole pregnancy (p < 0.01).
**Chuang** et al. **(2015) [**28**]** (cross-sectional study)	Participants were stratified in low (office workers) and high (welders) PM_2.5_ exposure with mean ± SD PM_2.5_ exposure levels of 27.4 ± 16.2 µg/m³ and 50.3 ± 32.8 µg/m³, respectively. The average PM_2.5_ concentrations of both office workers and welders exceeded the WHO air quality guidelines.	Welders with a mean ± SD age of 51.0 ± 9.7 years (n = 66) and office workers with a mean ± SD age of 48.2 ± 15.3 years (n = 12) working in a shipyard in southern Taiwan. Study period: 1 week	Levels of urinary KIM-1 and NGAL adjusted for urinary creatinine were significantly increased in welders post-exposure (p < 0.05), but no changes were observed in office workers post-exposure.
**Weaver** et al. **(2019) [**29**]** (cross-sectional study)	The mean ± SD 1-year and 3-year PM_2.5_ exposure levels were 12.2 ± 0.6 µg/m³ and 12.4 ± 0.5 µg/m³ respectively. The average PM_2.5_ concentration exceeded the WHO air quality guidelines.	African-Americans participating in the Jackson Heart Study (n = 5,090) with a mean ± SD age of 55.4 ± 12.8 years. Study period: 2000 – 2004	Inverse associations of 1-year and 3-year PM_2.5_ exposure could be observed with UACR (p < 0.05), indicating better a renal function with increasing PM_2.5_ exposure.
**Bernatsky** et al. **(2011) [**33**]** (cross-sectional study)	The average ± SD daily PM_2.5_ exposure was 10.0 ± 7.8 µg/m³ (range 1.1 – 54.9 µg/m³) PM_2.5,_ not exceeding the newly set WHO air quality guidelines.	Patients registered at the Lupus Clinic in Montreal (n = 237) with a mean ± SD age of 41.2 ± 15.5 years at the first visit. Study period: Jan. 2000 – Sept. 2007	No relationship between PM_2.5_ and SLEDAI-2 K scores could be demonstrated. Anti-dsDNA and urinary renal casts were significantly associated with PM_2.5_ exposure before the clinical visit(s) (p < 0.05); there was suggestive evidence of some association between anti-dsDNA and PM_2.5_ levels averaged over 10 days, although non-significant.
**Chen** et al. **(2018)** [38] (cross-sectional study)	Annual average ± SD PM_2.5_ concentration and PM_2.5_ absorbance amounted to 24.3 ± 3.9 µg/m³ and 1.8 ± 0.3 × 10^−5^/m, respectively. An IQR of 4.1 µg/m³ and 0.4 × 10^−5^/m was identified for PM_2.5_ exposure and PM_2.5_ absorbance, respectively. The average PM_2.5_ concentration exceeded the WHO air quality guidelines.	Elderly Taiwanese population with a mean ± SD age of 74.2 ± 6.5 years (n = 8,479). Of the total participants, 27.8% had CKD stage III to V (eGFR < 60 mL/min/1.73 m²). Study period: Mar. 2009 – Aug. 2009	A lower eGFR could be associated with one-year PM_2.5_ absorbance, but not PM_2.5_ exposure. For each IQR increment of PM_2.5_ absorbance proteinuria was non-significantly increased; no difference could be demonstrated for an IQR increment of PM_2.5_ exposure. A higher risk for CKD prevalence was demonstrated for PM_2.5_ and PM_2.5_ absorbance; the risk of CKD progression was elevated for PM_2.5_ absorbance.
**Wang** et al. **(2020) [**41**]** (cross-sectional study)	The mean ± SD PM_2.5_ exposure level was 61.22 ± 0.50 µg/m³, far exceeding the WHO air quality guidelines.	Hospitalized Chinese patients with a mean ± SD age of 60.37 ± 14.48 years (n = 3,622). Study period: Oct. 2014 – May 2015	No significant association could be shown between eGFR decline or CKD and PM_2.5_ exposure.
**Yang** et al. **(2017) [**42**]** (cross-sectional study)	The annual average ± SD PM_2.5_ exposure level was 26.64 ± 5.01 µg/m³, with an IQR of 5.67 µg/m³. The average PM_2.5_ concentration exceeded the WHO air quality guidelines. The annual average ± SD PM_2.5_ absorbance was 1.94 ± 0.39 × 10^−5^/m, with an IQR of 0.48 × 10^−5^/m.	Taiwanese citizens over 30 years of age with a mean ± SD age of 53.65 ± 10.37 years (n = 21,656). Of the total participants, 10.3% had CKD based on eGFR < 60 mL/min/1.73 m². Study period: 2007 – 2009	An IQR increment of PM_2.5_ exposure and/or PM_2.5_ absorbance indicated no association(s) with a lower eGFR or CKD.
**Liang** et al. **(2021)[**45**]** (nation-wide cross-sectional study)	The median (IQR) PM_2.5_ concentration amounted to 44.63 (18.65) µg/m³. The median exceeded by far the standard air quality guidelines set by the WHO.	Adults (>18 years old) residing in urban and rural areas in China (n = 47,086). The average age ± SD for participants with CKD (n = 4,790) and with no indications of CKD (n = 42,116) was 55.73 ± 16.37 and 48.90 ± 14.90 years, respectively. Study period: 2007 – 2010	Results indicated that elevated PM_2.5_ concentrations were significantly associated with increased odds of CKD prevalence. The results showed a stronger increase in odds for CKD in rural areas compared to urban areas (p_interaction_ < 0.001).
**Li G.** et al.**(2021)[**48**]** (cross-sectional study)	The mean ± SD 2-year PM_2.5_ concentration was 57.4 ± 15.6 µg/m³ (range: 31.3 to 87.5 µg/m³). The exposure levels exceeded by far the air quality guidelines set by the WHO.	Chinese adults (≥18 years; n = 47,204) with a mean ± SD age of 49.71 ± 15.54 and 49.47 ± 14.83 years for a mean ≤2 and >2-year PM_2.5_ concentration, respectively. Study period: Sept. 2009 – Sept. 2010	Each 10 µg/m³ increase in PM_2.5_ level was positively associated with CKD prevalence and albuminuria (p < 0.05). A significant difference was observed between urban and rural areas in CKD prevalence (p_interaction_ = 0.004).
**Kuźma** et al. **(2021)[**49**]** (retrospective cross-sectional study)	The median (IQR) PM_2.5_ concentration was 10.9 (15.9) µg/m³ during the entire study period. The PM_2.5_ levels exceeded the air quality guidelines set by the WHO.	Adults ≥18 years in China (n = 3,554) with a median age of 66 years. Study period: 2007 – 2016	With an increase in annual PM_2.5_ concentration, an increase in odds of CKD could be observed (p < 0.05). Furthermore, an IQR increase in weekly PM_2.5_ lead to a reduction in eGFR (p < 0.05).

Air quality guidelines for PM_2.5_ exposure by WHO in 2021 for daily and annual mean are 15 µg/m³ and 5 µg/m³, respectively [10]. The previous guidelines (2006) were 25 µg/m³ and 10 µg/m³, respectively [60]

*Abbreviations: CKD* chronic kidney disease, *dsDNA* double-stranded deoxyribonucleic acid, *eGFR* estimated glomerular filtration rate, *IQR* interquartile range, *KIM-1* kidney injury molecule-1, *NGAL* neutrophil gelatinase-associated lipocalin, *PM*_2.5_ fine particulate matter (<2.5 microns), *SD* standard deviation, *SLEDAI-2 K* systemic lupus erythematosus disease activity index version 2000, *UA* uric acid, *UACR* urinary albumin-to-creatinine ratio, *UN* urea nitrogen, *WHO* World Health Organization

**Table 3:** Data extraction of the 3 studies in the systematic review with a time-series or ecological study design

Authors (year) (type of study)	Exposure Estimate	Study Population	Main Findings
**Gu** et al. **(2020) [**32**]** (nation-wide time-series study)	The national average ± SD level of ambient PM_2.5_ exposure was 50.6 ± 18.2 µg/m³ during the study period, far exceeding the air quality guidelines set up by the WHO.	Hospital admissions from 252 Chinese cities were obtained from the Hospital Quality Monitoring System of China (n = 103,230,193). Study period: 2013 – 2017	Renal failure was significantly positively associated with PM_2.5_ exposure. Significant point estimates of the percentage increase (p < 0.001) in hospital admissions for nephritis, nephrosis, and renal sclerosis and chronic renal failure could be demonstrated in association with PM_2.5_ exposure.
**Bragg-Gresham ** et al. **(2018) [**36**]** (ecological study)	Exposure levels were allocated in low (≤12.2 µg/m³) and high (>12.2 µ/gm³) PM_2.5_ exposure levels (median PM_2.5_ = 12.2 µg/m³). Therefore, more than half of the exposure levels exceeded the WHO air quality guidelines. An IQR of 3.6 µg/m³ PM_2.5_ was identified.	Elderly population ≥65 years old (n = 1,164,057). The average age ± SD in the low and high PM_2.5_ exposure group was 75.4 ±7.7 and 75.2 ± 7.6 years, respectively. Study period: 2010	The overall prevalence of diagnosed CKD in the sample population was 17.2%. In unadjusted models, a higher prevalence ratio of diagnosed CKD with a 4 µg/m³ higher PM_2.5_ exposure was demonstrated. A higher prevalence of diagnosed CKD in more populated areas was observed compared to less densely populated areas (p < 0.0001).
**Bi** et al. **(2021)[**54**]** (time-series study)	The 24-hour average ± SD PM_2.5_ concentration was 15.41 ± 7.12 µg/m³, with an IQR of 8.99 µg/m³. The new daily air quality guidelines set by the WHO were slightly exceeded.	Persons that visited the emergency department (n = 306,595) for all renal diseases and acute renal failure.	Positive associations between short-term PM_2.5_ exposure and emergency room visits due to kidney disease outcomes and acute renal failure were observed per IQR increase.

Air quality guidelines for PM_2.5_ exposure by WHO (in 2021) for daily and annual mean are 15 µg/m³ and 5 µg/m³, respectively [10]. The previous guidelines (2006) were 25 µg/m³ and 10 µg/m³, respectively [60]

*Abbreviations*: *CKD* chronic kidney disease, *IQR* interquartile range, *PM*_2.5_ fine particulate matter (< 2.5 microns), *SD* standard deviation, *WHO* World Health Organization

Of all included studies, 19 studies [22–26, 31, 33–45, 47–53, 56, 58] had PM_2.5_ exposure levels that exceeded the old 2006 WHO air quality guidelines for ambient PM_2.5_, namely the annual mean (10 µg/m³) or the 24-hour mean (25 µg/m³) [60], of which 7 studies had PM_2.5_ exposure levels ranging from below the guideline to exceeding the pre-set guidelines [33, 34, 36, 44, 51, 56, 58]. Only 5 studies had a mean PM_2.5_ exposure level below the 2006 WHO air quality guidelines [27, 54, 55, 57, 59]. According to the new air quality guidelines announced in 2021, all studies exceeded the newly set guidelines for the annual mean (5 µg/m³) or the 24-hour mean (15 µg/m³) [10]. Additionally, 8 studies listed average PM_2.5_ exposure levels divergent from annual or daily means [15, 21, 24, 28–30, 32, 35, 46], i.e., 72-hour PM_2.5_ exposure means [21], various moving day averages [24] two-year [46], three-year [30] or seven-year [50] annual PM_2.5_ means, PM_2.5_ means during the study or follow-up period [32, 35], and means of PM_2.5_ exposure during the pregnancy [15].

Furthermore, 27 of the 40 included studies showed significant results or associations with investigated parameters and PM_2.5_ exposure [15, 21, 23, 26, 30–32, 34–37, 39, 40, 43–54, 58, 59]; 4 studies did not show any significant results or associations [24, 25, 42, 56]. It is of note that some articles (n = 9) [22, 27, 28, 33, 38, 40, 55, 57, 59] showed significant associations with one parameter, but not with another parameter investigated. Blum et al. [22] observed no significant association in eGFR, but did observe significant associations with an increased risk of incident CKD and higher levels of albuminuria. Gao et al. [27] showed that ambient PM_2.5_ exposure could be associated with a decline in eGFR, but not to serum uric acid, blood urea nitrogen or odds of developing CKD. Chang et al. [57] could not observe associations with higher odds of acute kidney rejection in the lowest quartile of PM_2.5_ exposure, but in could in the highest quartile. Chuang et al. [28] showed differences in urinary markers for welders post-exposure, but could not find significant differences in office workers post-exposure. Bernatsky et al. [33] showed no associations between PM_2.5_ exposure and the overall measurement to score systemic lupus erythematosus disease activity, but anti-double stranded DNA and urinary casts could be significantly associated to PM_2.5_ exposure. No differences in eGFR could be demonstrated for PM_2.5_ exposure, but Chen et al. [38] could for PM_2.5_ absorbance. Feng Y. and colleagues [55] could not show an association between mortality risk in dialysis patients and low levels of PM_2.5_ (<12 µg/m³), but did show associations for exposure to levels ≥12 µg/m³. Similar results were seen regarding PM_2.5_ exposure of kidney transplant recipients [59], where no association with one-year acute rejection could be shown below 12 µg/m³ PM_2.5_ exposure; though, associations were demonstrated with an increased risk of delayed graft function one-year acute rejection and all-cause mortality for each 10 µg/m³ increase in PM_2.5_ exposure. Lastly, Ran et al. [40] found that PM_2.5_ exposure was associated with renal failure mortality among hypertensive patients, but could not show associations with e.g., all-cause mortality among CKD patients.

## Discussion

### eGFR for Assessment of Renal Function

The estimated glomerular filtration rate (eGFR) describes the filtration of a certain volume of blood (milliliter) per unit of time (minutes) for a corporal surface of 1.73 m² by the glomerular capillaries into Bowman’s capsules. Despite that the gold standard for GFR evaluation is the measurement of inulin clearance, eGFR is currently used in medical practice to assess kidney function changes [61]. In the clinic, the GFR is estimated according to the Chronic Kidney Disease-Epidemiology Collaboration (CKD-EPI) equation. The single CKD-EPI equation for estimating GFR is eGFR = 141 × min(serum creatinine/κ, 1)^α^ × max(serum creatinine/κ, 1)^−1.209^ × 0.993^Age^ × 1.018 [if female] × 1.159 [if black], where serum creatinine is expressed in mg/dL, κ is 0.7 for females and 0.9 for males, and α is -0.329 for females and -0.411 for males [62]. Another method to estimate GFR, used commonly and addressed by various articles in this review is the Modification of Diet in Renal Disease (MDRD) equation: eGFR = 175 × (serum creatinine)^−1.154^ × (age)^−0.203^ × 0.742 [if female] × 1.212 [if black], where serum creatinine is expressed in mg/dL [63].

The effects of PM_2.5_ exposure on changes in eGFR was addressed in 12 of the included studies [15, 21–27, 42, 47, 49, 51]. These studies evaluating PM_2.5_ and eGFR have shown contradictory results. In the Atherosclerosis Risk in Communities cohort, participants were followed up from 1996 to 1998 to 2016 (mean age 63 years; n = 10,997); at baseline, no significant association between PM_2.5_ exposure and eGFR [eGFR = 0.07 mL/min/1.73 m², 95% confidence interval (CI) -0.28 to 0.41] was found [22]. In a cross-sectional study conducted on citizens (> 30 years; n = 21,656), Yang et al. [42] showed no significant association in eGFR decline (eGFR = -0.09 mL/min/1.73 m², 95% CI -0.25 to 0.07) for an interquartile range (IQR) increment of 5.67 µg/m³ in PM_2.5_. Furthermore, the latter report also did not show a significant association between change in eGFR (0.02 mL/min/1.73 m², 95% CI -0.16 to 0.19) and an IQR increment of 0.48 × 10^−5^/m in PM_2.5_ absorbance, which characterizes local soot emissions [42]. In a Flemish cohort (n = 820 at baseline participation and n = 653 at follow-up participation), Feng Y.M. et al. [25] stipulated that changes in eGFR [Odds ratio (OR)_baseline_ = 0.00, 95% CI -1.18 to 1.19; OR_follow−up_ = -0.30, 95% CI -1.78 to 1.18; and OR_combination_ = 0.01, 95% CI -1.16 to 1.17] or serum creatinine (OR_baseline_ = 0.23, 95% CI -1.07 to 1.52; OR_follow−up_ = 0.10, 95% CI -2.03 to 2.23; and OR_combination_ = 0.28, 95% CI -1.19 to 1.75) were unrelated to PM_2.5_ exposure (p ≥ 0.69) when considering only the baseline exposure levels, only the follow-up, or a combination. Lastly, Li A. et al. [24] investigated the effects of PM_2.5_ exposure on eGFR in a population (n = 169) residing in China. A decline in eGFR, calculated through both the MDRD (e.g., 60-day exposure: 2.78%, 95% CI -4.61 to 10.73) and CKD-EPI (e.g., 60-day exposure: 1.05%, 95% CI -2.51 to 4.75) equations, was inversely associated with each IQR increase in PM_2.5_ concentrations, albeit nonsignificant (p > 0.05).

However, in a prospective cohort study on young adults aged 18 to 45 years (n = 2,546,047) of Han ethnicity, Li Q. et al. [26] observed a 0.77% decrease in eGFR (95% CI -0.81 to -0.73; p < 0.05) for each 10 µg/m³ increment in PM_2.5_ exposure. Additionally, for each 10 µg/m³ PM_2.5_ increment, a significant increase of 0.87% (95% CI 0.82 to 0.91; p < 0.05) in serum creatinine could be shown [26]. Furthermore, associations between eGFR or serum creatinine and PM_2.5_ exposure levels were higher in females than in males (p < 0.05). In another a prospective cohort study on war veterans (mean age 73.5 years at the first visit; n = 669), Mehta et al. [23] showed that a 2.1 µg/m³ higher one-year PM_2.5_ exposure was significantly associated (p < 0.05) with a 1.87 mL/min/1.73 m² lower eGFR (95% CI -2.99 to -0.76); additionally, the aforementioned increment in one-year PM_2.5_ was significantly associated (p < 0.05) with an annual decrease in eGFR of 0.60 mL/min/1.73 m² (95% CI -0.79 to -0.40). They could not only link PM_2.5_ to a reduced eGFR, but also to an increased rate of eGFR decline over time [23]. A second study [27] performed on this population assessed the short-term (28-day window) effects of PM_2.5_ exposure on renal function. The average ± SD 28-day PM_2.5_ concentration was 9.27 µg/m³ ± 3.08 µg/m³ to which the 808 elderly war veterans with a mean ± SD age of 75.7 ± 7.2 years (n = 2,466 study visits over those 808 participants) were exposed. Results indicated a robust association between PM_2.5_ exposure and lower eGFR. For each 4.09 µg/m³ increment in 28-day PM_2.5_, a mean ± standard error (SE) change of -1.6078 ± 0.4446 mL/min/1.73 m² was observed (p < 0.001).

A retrospective cross-sectional study by Kuźma et al. [49] on patients, referred for elective coronary angiography (n = 3,554) with a median age of 66 years, showed that a 15.9 µg/m³ increase in weekly PM_2.5_ exposure was associated with a 2% reduction in expected eGFR (β = 0.02, 95% CI -0.03 to -0.01; p < 0.05). In another cross-sectional study design on pregnant women (n = 10,052), Zhao et al. [15] estimated a significant reduction (p < 0.01) in eGFR of 0.54 mL/min/1.73 m² (95% CI -0.74 to -0.33) during the entire pregnancy for an IQR increment of PM_2.5_. In a prospective war veterans study (n = 2,482,737), Bowe et al. [51] reported an increased risk (p ≤ 0.05) of incident eGFR <60 mL/min/1.73 m² [hazard ratio (HR) = 1.25, 95% CI 1.17 to 1.34] for a 10 µg/m³ increment in PM_2.5_. Compared to participants exposed to lower PM_2.5_ levels (5.0 – 9.1 µg/m³), participants exposed to higher PM_2.5_ levels (> 9.2 µg/m³) had a gradually increased risk of incident eGFR < 60 mL/min/1.73 m [51]. In addition, in a general adult Taiwanese population (n = 108,615) with an average ± SD age of 39.1 ± 11.8 years, Zeng et al. [47] demonstrated that each 10 µg/m³ increment of PM_2.5_ could be significantly associated (p < 0.001) with a 3.18-fold increased risk of an eGFR decline ≥30% (95% CI 2.88 to 3.50). In an elderly population (60 – 69 years old; n = 71), the panel study of Fang et al. [21] showed that an IQR increment of 31.43 µg/m³ in PM_2.5_ exposure was significantly associated (p < 0.05) with a 3.27% eGFR decrease, albeit in a small population size (n = 71).

The underlying mechanism(s) that may explain why 4 studies [22, 24, 25, 42] showed no association, while 8 other studies [15, 21, 23, 26, 27, 47, 49, 51] did show associations between a decline in eGFR and PM_2.5_ exposure, remain unexplored. Yet, as PM_2.5_ components vary by region, certain regions may contain higher concentrations of such components that are more detrimental to glomerular and proximal tubular function leading to differences in eGFR measurements [15, 22, 23]. However, it is of note that Mehta et al. [23], Gao et al. [27], Zhao et al. [15], and Bowe et al. [51] investigated overall kidney function on distinctive subpopulations, i.e., elderly veterans with a mean age of 73.5 years [23] and 75.7 years [27] respectively, Chinese pregnant women [15], and veterans with a median age of 62.5 years [51], which are not representative for the general population, in contrast to the studies by Fang et al. [21], Blum et al. [22], Feng Y.M. et al. [25], Li A. et al. [24], Li Q. et al. [26], Kuźma et al. [49], Zeng et al. [47], and Yang et al. [42]. In addition, the GFR in normal pregnancies can increase 40 to 50%; therefore, the current standard in clinical practices to evaluate glomerular filtration rate is the creatinine clearance in 24-hour urine [64]. Zhao et al. [15] measured serum creatinine but did not describe any changes in creatinine clearance in relation to PM_2.5_ exposure.

It is of note that not all studies use the same equation to determine eGFR. Of all studies addressing measurement(s) of eGFR in this review, 5 studies [21, 43, 46–48] estimated GFR according to the MDRD equation, one study [51] did not specify, and one study [24] used both equations to estimate GFR, while 13 studies [22, 23, 25–27, 29, 34, 37, 38, 41, 42, 44, 49] used the CKD-EPI equation to determine eGFR. Current research is contradictory about the superiority of either methods to estimate GFR [38, 62, 65, 66]. However, in 2013, the CKD-EPI equation was recommended by the ‘Kidney Disease: Improving Global Outcomes (KDIGO) to estimate GFR [67].

### Evaluation of Renal Function through Biomarkers in Urine and Blood

In addition to the use of eGFR measurements and creatinine clearance in the routine assessment of kidney function, urinary biomarkers are progressively used as a noninvasive tool to evaluate the glomerular and tubular functions of the kidneys.

Albuminuria is a sensitive, prognostic marker for changes that are observed in the glomerulus and its permeability to macromolecules [68]. Five studies focused on albuminuria after PM_2.5_ exposure [22, 24, 25, 29, 48]. Li G. et al. [48] demonstrated that each 10 µg/m³ increment of PM_2.5_ exposure could be positively associated (OR = 1.39, 95% CI 1.32 to 1.47) with albuminuria (p < 0.001) in an adult Chinese population (n = 47,204). The study by Blum and colleagues [22] showed that a higher annual average PM_2.5_ exposure was associated with increased albuminuria. They concluded that a 1 µg/m³ increment of the annual average PM_2.5_ was significantly associated (p ≤ 0.001) with a higher urinary albumin-creatinine ratio (UACR) (6.6% difference, 95% CI 2.6 to 10.7%) [22]. The cross-sectional study design of Weaver et al. [29] has shown opposite results, including inverse associations (p < 0.05) of UACR with 1-year and 3-year averaged PM_2.5_ exposure (OR = -0.09, 95% CI -0.2 to -0.02 and OR = -0.2, 95% CI -0.3 to -0.06, respectively). The authors stated that this may be due to bias, since participants with better kidney function are more likely to provide urine samples when operating on a voluntary basis [29]. The study by Feng Y.M. et al. [25] showed that changes in microalbuminuria (OR_baseline_ = 0.27, 95% CI -0.26 to 0.79; OR_follow−up_ = 0.06, 95% CI -0.43 to 0.54; and OR_combination_= 0.21, 95% CI -0.19 to 0.61) were unrelated to PM_2.5_ exposure (p ≥ 0.31) when considering only the baseline, only the follow-up, or a combination of participation (n = 820 at the baseline participation and n = 653 at follow-up participation). Furthermore, in a small population sample (n = 169) of participants with an average ± SD age of 64.0 ± 8.7 years, Li A. et al. [24] could not show any associations between an IQR increase in PM_2.5_ and UACR (e.*g.*, 60-day exposure: 0.05%, 95% CI -0.50 to 0.61; p > 0.05).

Other promising markers include urinary kidney injury molecule-1 (KIM-1) [69] and neutrophil gelatinase-associated lipocalin (NGAL) [28, 70], tubular markers of extracellular matrix receptor interaction [28, 70]. Using the urinary markers KIM-1 and NGAL, Chuang et al. [28] investigated the renal effects of exposure in welders and office workers in a cross-sectional study of whom the personal PM_2.5_ exposure (50.3 µg/m³ and 27.4 µg/m³, respectively) exceeded the daily guideline set by the WHO at that time frame (25 µg/m³) [60]. Urinary levels of KIM-1 were significantly increased in welders post-exposure compared to pre-exposure (p < 0.05), but no difference was observed between post- and pre-exposure in office workers. Urinary NGAL was significantly higher (p < 0.05) in welders as well as office workers post-exposure compared to pre-exposure. These results indicate that PM_2.5_ might negatively affect tubular kidney function during short periods of exposure (one working week between pre- and post-exposure measurements of the biomarkers) [28].

Other traditional biomarkers employed in medical practice include measurements of uric acid (UA) and urea nitrogen (UN) in blood. Increases in UA or UN are an indication of kidney function decline, due to a decrease in the glomerular filtration rate [15, 71]. In pregnant women, Zhao et al. [15] demonstrated for a IQR increment of 3.90 µg/m³ PM_2.5_ exposure increases of 0.05 mmol/L (95% CI 0.04 to 0.07) in serum UN and 0.60 mmol/L (95% CI -0.86 to 2.06) in serum UA, indicating a reduction of eGFR. Whereas serum UN was found to be significantly positively associated (p < 0.01) with PM_2.5_ during the whole pregnancy, no such association could be shown for serum UA [15]. Furthermore, Gao et al. [27] indicated that no significant associations (p > 0.06) could be shown for UA (0.0674 mg/dL; SE: 0.0442 mg/dL) and UN (0.0110 mg/dL; SE: 0.1987 mg/dL) for each 4.09 µg/m³ increase in short-term 28-day PM_2.5_ concentration.

The use of novel renal biomarkers in relation to PM_2.5_ exposure in a clinical setting remains largely underexplored. Various other well-documented urinary glomerular markers such as cystatin C [72] and urinary tubular markers such as β_2_-microglobulin [73], α_1_-microglobulin [74], and retinol-binding protein [75] have not been investigated in (large) population-based studies in relation to the early toxic effects of environmental PM_2.5_ exposure on kidney function. Further research is required to determine their potential both in research and clinical settings.

### Glomerular Diseases

Glomerular diseases (e.g., glomerulonephritis, such as nephrotic syndrome) are caused by damage to the glomeruli, which may cause leakage of large proteins into the urine and interference with the clearance of waste products, which can result in a buildup of harmful substances in the blood. Four of the included studies addressed the effects of PM_2.5_ exposure on the glomeruli [30–32], of which one specifically addressed the auto-immune disorder systemic lupus erythematosus (SLE) [33].

In a time-series study on hospital admissions attributable to nephritis [32], a 10 µg/m³ increment in PM_2.5_ exposure caused a significant percental change of 0.23 (95% CI 0.08 to 0.39; p < 0.05) in hospital admissions due to nephritis. The cohort study by Lin S.Y. et al. [31] demonstrated a clear dose-response relationship between PM_2.5_ exposure and nephrotic syndrome (NS) in a population cohort of residents of Taiwan (n =161,970). An increasing trend for risk of developing NS was shown; relative to the lowest PM_2.5_ exposure level (quartile 1; < 29.5 µg/m³), the highest level of PM_2.5_ exposure (quartile 4; > 41.2 µg/m³) was associated with a 2.53-fold higher risk of developing NS (HR = 2.53, 95% CI 1.01 to 5.94; p < 0.05) [31].

A common cause of NS in adults is idiopathic membranous nephropathy (iMN), where the immune system attacks the glomeruli, leading to kidney damage [76]. A cohort study by Xu et al. [30] demonstrated that higher PM_2.5_ exposure was associated with an increased risk of iMN. However, the relationship appeared non-linear below PM_2.5_ concentrations of 70 µg/m³; above 70 µg/m³, an increment of 10 µg/m³ PM_2.5_ exposure was associated with 14% higher odds to develop iMN (95% CI 1.10 to 1.18). The frequency of iMN varied among geographical regions, with the most polluted areas having the highest frequency [30].

All three studies [30–32] addressing glomerular diseases associated with PM_2.5_ exposure showed that PM_2.5_ negatively affects the glomeruli. However, the mechanisms by which PM_2.5_ exposure triggers or exacerbates glomerular diseases remain unknown, requiring more research to elucidate this association. It would be worthwhile to explore whether glomerular diseases presumably linked with a direct toxic insult of PM_2.5_ exposure on the glomeruli could not be the result of an indirect harmful influence on the glomerular function associated with the progression of tubulointerstitial changes secondary to PM_2.5_ exposure-linked tubular lesions. The pathophysiological mechanisms of NS development are believed to be associated with autoimmunity that targets the glomerulus. Air pollution and PM_2.5_ exposure have recently been associated with alterations in autoimmunity, leading to increases in oxidative stress and inflammation, which may trigger autoimmune reactions [31, 77, 78].

Auto-immune diseases, such as SLE, may be associated with varying PM_2.5_ exposure. In a cross-sectional study design, Bernatsky et al. [33] investigated whether PM_2.5_ exposure affected clinical aspects of SLE, listed in the SLE Disease Activity Index version 2000 (SLEDAI-2 K). No clear-cut association between PM_2.5_ exposure and overall SLEDAI-2 K scores could be demonstrated. The authors stipulated that features of disease activity could have been present early in the 10-day window preceding the assessment but were not present at the time of the hospital visit [33]. However, urinary casts – which reflect renal inflammation – and anti-dsDNA were associated with short-term variations in PM_2.5_ exposure shortly before the clinical visits (24 to 48 h before). A 10 µg/m³ increment in PM_2.5_ exposure was significantly associated (p < 0.05) with increased odds for the presence of urinary casts (1.43, 95% CI 1.05 to 1.95) as well as the presence of anti-dsDNA (1.26, 95% CI 0.96 to 1.65) [33]. PM_2.5_ exposure could not be associated with clinical disease activity features of SLE. These results suggest that PM_2.5_ exposure has acute effects on the occurrence of anti-dsDNA and urinary casts; however, as the kinetics of anti-dsDNA and urinary casts are not entirely understood, it is physiologically plausible that these markers vary daily with fluctuating PM_2.5_ exposure.

### Diabetes Mellitus as a Driver of Kidney Function Decline

Exposure to PM_2.5_ has been shown to exacerbate pre-existing health conditions, such as diabetes, in both humans and animals [79–81]. Diabetes causes damage to the kidneys by narrowing of the afferent and efferent arterioles transporting blood to and from the kidneys [82, 83]. Therefore, the presence of a pre-existing disease might act as a mediator to adverse kidney outcome(s) following PM_2.5_ exposure, exacerbating the decline of kidney function.

Diabetes mellitus is a significant risk factor for kidney damage, as blood vessels in the kidney become damaged and high blood pressure will occur. The buildup of water and salts leads to hyperfiltration, causing harm to the nephrons and excess proteinuria [83]. Two studies investigated diabetes mellitus as a mediator of kidney function decline after PM_2.5_ exposure [34, 35]. A cohort study conducted by Chin and colleagues [35] showed that patients exposed to lower levels of PM_2.5_ (quartile 1; 27.7 µg/m³) and those exposed to higher levels of PM_2.5_ (quartile 3; 38.8 µg/m³) had an annual UACR increase of 3.17 mg/g and 3.96 mg/g respectively. A more rapid progression of microalbuminuria (20 – 200 mg/L) in patients exposed to higher levels of PM_2.5_ was observed [35], which may be explained by the known effects of PM_2.5_ on increased insulin resistance [84]. Additionally, Bowe et al. [34] indicated diabetes as a mediator in the association of PM_2.5_ exposure with kidney disease outcomes in an prospective cohort study, where a 10 µg/m³ increment in PM_2.5_ exposure was associated with increased odds of diabetes (OR = 1.18, 95% CI 1.06 to 1.32), and also with on average a 1.2-fold increased risk of kidney disease outcomes. However, the proportions of association between PM_2.5_ exposure and kidney disease outcomes mediated by having diabetes varied: 4.7% (95% CI 4.3 to 5.7%) for incident eGFR < 60 mL/min/1.73 m², 4.8% (95% CI 4.2 to 5.8%) for incident CKD, 5.8% (95% CI 5.0 to 7.0%) for ≥ 30% decline in eGFR, and 17.0% (95% CI 13.1 to 20.4%) for ESRD or ≥ 50% decline in eGFR [34]. The study showed that only a small proportion (< 6%) of the association between PM_2.5_ exposure and the risk of kidney disease outcomes is mediated by diabetes, except for ESRD. The mediation effect of diabetes is much higher for ESRD, likely due to the rapid progression of CKD to ESRD because of diabetes, which may reflect from the relative strength of the association between diabetes and ESRD in comparison to other investigated kidney diseases. However, it is of note that the study conducted by Bowe et al. [34] may not be generalizable to the general population as the cohort only included United States war veterans with a mean age of 62.5 years. Lastly, Feng Y. et al. [55] indicated that when diabetes was the primary cause of kidney failure (aHR = 1.25, 95% CI 1.13 to 1.38), patients were more vulnerable to high PM_2.5_ exposure levels with an increased mortality risk.

The occurrence of a pre-existing driver such as diabetes mellitus might exacerbate the effects that PM_2.5_ exposure exerts on the kidneys and its functioning. The sparse studies indicated that diabetes mellitus might act as a mediator between kidney disease outcomes (e.g., CKD, ESRD) and PM_2.5_ exposure.

### Chronic Kidney Disease

Diabetes is a significant cause of CKD [85], due to a gradual loss of kidney function over a period of months to years. The development and progression of CKD is associated with an increasing all-cause mortality [86]. The CKD-linked disability-adjusted life-years (DALYs) attributable to PM_2.5_ globally in 2016 have been estimated at 11.4 million years [4]. There is no consensus yet about the adverse effects of PM_2.5_ on CKD development and progression. Of all included studies, 17 studies focused on the effects of PM_2.5_ exposure on CKD [22, 27, 36–49, 51].

Cross-sectional studies conducted by Wang et al. [41] and Yang et al. [42] demonstrated the lack of significant associations between PM_2.5_ exposure and lower eGFR as an indication of CKD development (β = 0.10, 95% CI -0.30 – 0.49 [41] and β = -0.09, 95% CI -0.25 to 0.07 [42], respectively) as well as increased CKD prevalence (OR = 0.99, 95% CI 0.91 to 1.08 [41] and OR = 1.03, 95% CI 0.97 to 1.09 [42], respectively). However, Wang et al. [41] stipulated that not all crucial confounders were estimated and taken into account in the statistical model(s), which might also be a limitation in the study conducted by Yang and colleagues [42]. Another cross-sectional study, investigating an elderly population (≥ 65 years of age) with stage III to V of CKD, could not show any associations between eGFR as a measure for CKD development or progression and a 4.1 µg/m³ IQR increment of PM_2.5_ exposure [38]. Of all subjects, 62.8% had hypertension and 17.5% had diabetes at the moment of inclusion in the study. Persons were categorized as having low (68.3%), moderate (20.2%), high (7.2%), or very high risk (4.3%) of CKD progression. For the total population (n = 8,497), the percental changes of eGFR and eGFR < 60 mL/min/1.73 m² were 0.24 (95% CI -0.19 to 0.67) and 0.54 (95% CI -0.21 to 1.26), respectively [38]. In the cohort study by Feng Y.M. et al. [25], focusing on white Europeans (n = 820 at the baseline participation and n = 653 at follow-up) with an average ± SD age of 51.1 ± 15.6 years, the authors observed that changes in CKD stage(s) (OR_baseline_ = -0.09, 95% CI -0.42 to 0.24; OR_follow-up_ = 0.04, 95% CI -0.29 to 0.38; and OR_combination_ = -0.01, 95% CI -0.29 to 0.26) were unrelated to PM_2.5_ exposure (p ≥ 0.59) when considering only the baseline, only the follow-up, or a combination of participation.

The cross-sectional study by Chen et al. [38] also evaluated PM_2.5_ absorbance, which characterizes local soot emissions. For an IQR increment of PM_2.5_ absorbance of 0.4 × 10^-5^/m a significant association was observed with a lower eGFR (-1.07% change, 95% CI -1.57% to -0.54%), higher CKD prevalence (OR = 1.126, 95% CI 1.057 to 1.199), and CKD progression (OR = 1.114, 95% CI 1.051 to 1.181) [38]. These results indicate that emission sources, such as from industrial or residential activities, can contribute to airborne PM and locally change the air pollution composition [38, 87].

Ghazi et al. [44] investigated CKD prevalence on adult patients (n = 113,725), where for each 1 µg/m³ increase in PM_2.5_, no association could be demonstrated with CKD prevalence when CKD was defined as having an eGFR <60 mL/min/1.73 m²; however, when CKD was defined as having an eGFR <45 mL/min/1.73 m², the highest quartile (Q4 ≥10.7 µg/m³) of PM_2.5_ was associated with greater odds of CKD (OR = 1.18, 95% CI 1.05 to 1.33) compared to the lowest quartile (Q1 <9.5 µg/m³). Additionally, each 1 µg/m³ increase in PM_2.5_ concentration could be associated with 7% greater odds of CKD (95% CI 1.03 to 1.11). At baseline, 35%, 11%, and 9% of patients had hypertension, diabetes, and/or cardiovascular disease(s), respectively.

Li G. et al. [48] demonstrated that with each increase of 10 µg/m³ PM_2.5_, a positive association (p < 0.001) could be observed with CKD prevalence (OR = 1.28, 95% CI 1.22 to 1.35) in an adult Chinese population. In this nation-wide cross-sectional study (n = 47,086), a 10 µg/m³ increment in PM_2.5_ was shown to be significantly associated with increased odds for CKD (OR = 1.24, 95% CI 1.14 to 1.35) at 1- to 4-year moving averages of PM_2.5_ exposure (p < 0.001). Furthermore, stronger increased odds for CKD was demonstrated in rural areas (OR = 1.51, 95% CI 1.29 to 1.78) compared to urban areas (OR = 0.92, 95% CI 0.79 to 1.07) per increase of 10 µg/m³ PM_2.5_ at 2-year moving average (p_interaction_ < 0.001) [45]. In the Veterans Affair Normative Aging cohort study by Gao et al. [27], for each 4.09 µg/m³ increment in short-term (28-day) PM_2.5_ concentration, an increased odds for CKD (OR = 1.1399, 95% CI -0.0099 to 0.2718) was observed, albeit non-significant (p > 0.05). Kuźma et al. [49] performed a retrospective cross-sectional study and showed that with each 15.9 µg/m³ increment in annual PM_2.5_ exposure, the odds of CKD significantly increased (OR = 1.07, 95% CI 1.01 to 1.15; p = 0.037). Additionally, in a population of 2,482,737 users of the Veterans Affairs Healthcare System, with median of 8.52 years of follow-up, Bowe et al. [51] observed an increased risk of incident CKD (HR = 1.27, 95% CI 1.17 to 1.38) for a 10 µg/m³ increment in annual average PM_2.5_ exposure, with an elevated risk at PM_2.5_ concentrations > 9.2 µg/m³ (p ≤ 0.05). Of the overall cohort, 67.26%, 27.80%, and 29.86% of persons had a diagnosis of hypertension, diabetes, and/or cardiovascular disease(s), respectively. In a prospective follow-up of 10 years, Blum et al. [22] reached similar conclusions, in that the annual average of PM_2.5_ exposure in four counties (range: 9.4 – 15.3 µg/m³) was significantly associated with an increased risk of CKD (HR = 1.05, 95% CI 1.01 to 1.10; p < 0.05). Here, 16%, 46%, and 19% participants exposed to lower or equal to the site-specific median (range: 9.4 – 15.3 µg/m³) had diabetes, hypertension, and composite cardiovascular disease, respectively. Comorbidity was similar for participants exposed to a higher site-specific median (17% for diabetes, 49% for hypertension, and 20% for composite cardiovascular disease). Furthermore, a population-based longitudinal cohort study conducted in Taiwan [37] showed that a higher level of PM_2.5_ exposure was associated with a higher risk of developing CKD. At the baseline of the study, 16.0% and 5.0% of participants had a diagnosis of hypertension or diabetes, respectively [88]. Relative to the participants exposed to low PM_2.5_ exposure levels (5.8 – 21.1 µg/m³), those exposed to higher PM_2.5_ levels (>25.5 µg/m³) had a significantly increased risk of developing CKD (HR = 1.15, 95% CI 1.05 to 1.26; p < 0.05) [37]. For a 10 µg/m³ increment of PM_2.5_ exposure, Chan et al. [37] reported a significant risk of CKD incidence (HR = 1.06, 95% CI 1.02 to 1.10; p < 0.05). In another longitudinal cohort study also evaluating the general Taiwanese population (n = 104,092) with a follow-up ≥ 3 years, each increment of 10 µg/m³ PM_2.5_ could be significantly associated with a 2.66-fold (95% CI 2.43 to 2.90) increased risk of developing CKD (p < 0.001) [47]. Ghazi et al. [44] also investigated CKD incidence in the adult patient population with no CKD at baseline (n = 20,289) and observed that each 1 µg/m³ increase in baseline annual average PM_2.5_ was associated with an increased risk of CKD (HR = 1.78, 95% CI 1.65 to 1.89; p < 0.05). Increasing risk was demonstrated for increasing quartiles of baseline PM_2.5_ exposure, with an HR of 1.72, 2.15, and 2.49 for the second (Q2 9.5 – 10.1 µg/m³), third (Q3 10.1 to 10.7 µg/m³) and fourth (Q4 ≥10.7 µg/m³) quartiles of PM_2.5_ exposure compared to quartile one (Q1 <9.5 µg/³) (p < 0.05).

A third nation-wide Taiwanese study by Lin S.Y. et al. [39] reported that exposure levels of PM_2.5_ above 28.3 µg/m³ (quartile 1) were associated with increased hazard ratios of CKD risk (p < 0.001). They showed an increased risk of CKD incidence for a 1 µg/m³ increment in PM_2.5_ exposure (HR = 1.03, 95% CI 1.02 to 1.03) [39]. It is of note that 29.1%, 10.1%, and 14.1% of participants had hypertension, diabetes, and coronary artery disease at the time of measurements. In an ecological study by Bragg-Gresham et al. [36] addressing the elderly population (age ≥65 years), 28.6% and 73.2% of the participants in the low (≤ 12.2 µg/m³) PM_2.5_ exposure group had a diagnosis of diabetes and hypertension respectively, 32.2% and 77.4% were diagnosed with diabetes and hypertension respectively in the high PM_2.5_ exposure group (> 12.2 µg/m³). For all participants in their Medicare population cohort (n = 1,164,057), the study showed a prevalence ratio of 1.03 (95% CI 1.02 to 1.05) of diagnosed CKD in association with a 4 µg/m³ increment of PM_2.5_ exposure. However, it is of note that this positive association between PM_2.5_ exposure and diagnosed CKD was shown in an elderly population; hence, these results may not be generalizable for younger (sub)populations.

Ran et al. [40] investigated the mortality risk of ambient PM_2.5_ exposure on cause-specific mortality in CKD patients in a prospective study design, where 67.0% and 34.9% had self-reported diabetes and hypertension, respectively. They reported an adjusted HR of 1.13 (95% CI 0.98 to 1.30) on all-cause mortality. Furthermore, a 4.0 µg/m³ IQR increment of PM_2.5_ was associated with mortality from CKD progression (HR = 1.17, 95% CI 0.89 to 1.53) [40]. In a retrospective cohort study by Jung et al. [43], CKD patients were followed up to evaluate mortality risk after PM_2.5_ exposure. Of these CKD patients, 21% and 36% reportedly had diabetes and hypertension, respectively. The authors observed that CKD patients who survived were exposed to lower levels of PM_2.5_ exposure than the CKD patients who passed away during the study period (p < 0.001). For each 2.55 µg/m³ IQR increment in PM_2.5_ concentration, a significantly increased risk of mortality was observed (HR = 1.17, 95% CI 1.07 to 1.29; p = 0.019). Therefore, PM_2.5_ exposure may not only play a crucial role in the worsening of CKD, but may also contribute to circulatory damage and be involved in a synergistic effect between hypertension and PM_2.5_ exposure, which may accelerate CKD progression into renal failure [40, 89].

In a longitudinal cohort study by Bo and colleagues [46], 163,197 participants were followed up for an average of 5.1 years (range 1.0 to 15.9 years). Of participants, 12.7% and 3.3% had hypertension and diabetes, respectively. Bo et al. [46] stipulated that for each 5 µg/m³ decrease in PM_2.5_ levels, positive associations were found for incident CKD (HR = 0.75, 95% CI 0.73 to 0.78). They concluded that a lower risk of CKD development could be associated with chronic PM_2.5_ exposure improvement. This is the first study to suggest that reducing air pollution may be an effective strategy for the prevention of CKD.

It is important to note that out of seventeen studies, only the studies by Chen et al. [38] and Feng Y.M. et al. [25] addressed stages of CKD that were included in their analysis; all other studies did not. Ran et al. [40] suggested that CKD patients enrolled in their study could be in the moderate to high stages, as symptoms were severe enough to be hospitalized. Furthermore, not every study addressed significant drivers of the development and progression of CKD, such as hypertension [22, 36–40, 43–49, 51], diabetes [22, 36–40, 43–48, 51], and cardiovascular disease [22, 39, 44, 45, 47, 48, 51].

Of the seventeen studies [22, 27, 36–49, 51] evaluating the association between PM_2.5_ exposure and CKD, five could not show any associations [25, 38, 41, 42, 44]; however, the evidence showing an effect of ambient PM_2.5_ exposure on CKD development, incidence, prevalence, and mortality is rising. As the global burden of CKD attributable to PM_2.5_ exposure is significant, efforts to improve ambient air quality are necessary to mitigate this burden [4, 51].

### End-Stage Renal Disease and Kidney Failure

CKD may gradually worsen into an advanced stage, termed end-stage renal disease (ESRD), requiring invasive treatments, such as dialysis or kidney transplantation, to maintain quality of life. Even though the prevalence and incidence of ESRD rise globally [50], the association with PM_2.5_ exposure remains to be elucidated [50, 51]. Of the included studies, the effects of PM_2.5_ on the (risk of) development of ESRD was addressed in 3 studies [39, 50, 51].

Relative to low PM_2.5_ exposure levels (Q1 ≤ 11.71 – 28.69 µg/m³), Wu et al. [50] found for patients exposed to higher exposure levels (Q4 39.96 – 46.63 µg/m³), an increased risk of developing ESRD (HR = 1.15, 95% CI 1.01 to 1.30; p < 0.05) and also a higher cumulative incidence of ESRD, compared to patients exposed to lower PM_2.5_ levels (<39.96 µg/m³), in a prospective study design. An IQR increment of 11.31 µg/m³ in PM_2.5_ exposure was associated with a 8% elevated risk of developing ESRD (95% CI 1.00 to 1.15) [50]. Bowe et al. [51] reported that for a 10 µg/m³ increment in PM_2.5_ the risk of developing ESRD was 1.31 (95% CI 1.21 to 1.43)  Per 100,000 person-years, the incident rate of ESRD was 44.36 (95% CI 44.27 to 44.45) with higher incidence rates for increasing PM_2.5_ levels (p ≤ 0.05). Another prospective nation-wide cohort study reported similar results, in that ESRD risk increased for an increment of 1 µg/m³ daily average PM_2.5_ exposure (aHR = 1.02, 95% CI 1.01 to 1.03; p ≤ 0.01) [39]. The risk of developing ESRD was more elevated for higher levels of PM_2.5_ exposure (>34.0 µg/m³), relative to participants exposed to lower PM_2.5_ concentrations (<28.3 µg/m³) [39].

When components of the kidneys, such as the glomeruli, the tubules, or the tubule-interstitium are damaged, CKD may develop and rapidly progress into ESRD [90]. The hypothesis emerging from recent studies that the development of CKD may be causally linked to air pollution, and more specifically to PM_2.5_ exposure, requires more research to unravel the mechanisms of PM_2.5_ involvement in the development of CKD and its rapid progression and/or exacerbation into ESRD [4, 39].

Progression of CKD could also lead to kidney failure, the progressive loss of kidney function. It is believed that the prevalence of kidney failure and the need for replacement therapy will double in the next years, leading to substantial socioeconomic costs [52, 53]. Six studies investigated the effects of PM_2.5_ exposure on the development, progression, and visits to the emergency room because of mortality from renal failure [32, 40, 52–55].

In a time-series study by Bi et al. [54], positive associations could be observed between short-term exposure to PM_2.5_ (8-day) and emergency room visits due to acute renal failure [relative risk (RR) = 1.026, 95% CI 0.997 to 1.057] per IQR (8.99 µg/m³) increase of PM_2.5_ exposure. Overall, results showed that exposure to PM_2.5_ for a longer period was associated with a higher risk of kidney disease outcomes. Furthermore, another time-series conducted by Gu et al. [32] investigated the number of hospital admissions attributed to kidney failure. Not only was kidney failure shown to be significantly positively associated with same-day PM_2.5_ exposure, but a 10 µg/m³ increment of PM_2.5_ exposure was associated (p < 0.001) with a 0.32% change in hospital admissions (95% CI 0.19 to 0.45) attributable to kidney failure.

In a cohort study by Feng Y. et al. [55], the authors showed that for older patients (≥65 years) on their first-time maintenance dialysis, a 10 µg/m³ increase in PM_2.5_ concentration could be associated with a 1.16-fold (95% CI 1.08 to 1.25) increased risk of mortality. Furthermore, these associations were stronger at higher levels (>12 µg/m³) of PM_2.5_ (aHR = 1.19, 95% CI 1.08 to 1.32), but were still significantly associated at lower levels (≤12 µg/m³) of PM_2.5_ with mortality risk (aHR = 1.04, 95% CI 1.00 – 1.07).

Ran et al. [40] showed that a 4.0 µg/m³ IQR increment of PM_2.5_ exposure was associated with an increased risk of renal failure mortality in CKD patients (HR = 1.18, 95% CI 0.91 to 1.52) and CKD patients with existing hypertension (HR = 1.42, 95% CI 1.05 to 1.93). Moreover, CKD patients with hypertension had a significantly higher risk of renal failure mortality (HR = 1.42, 95% CI 1.05 to 1.93; p < 0.05). A retrospective study by Ran et al. [52] investigated whether the risk of kidney failure mortality differed between a cohort of the general elderly population (≥ 65 years of age; n = 61,447) and patients diagnosed with CKD. They showed that a 3.22 µg/m³ IQR increment of PM_2.5_ exposure was associated with increased mortality risk in both the cohort participants (HR = 1.23, 95% CI 1.06 to 1.43; p < 0.01) and the patients diagnosed with CKD (HR = 1.42, 95% CI 1.16 to 1.74; p ≤ 0.001). The subcategory analysis of renal failure, including the development of all incidence cases of acute kidney injury and CKD, also showed significant associations (p ≤ 0.001) with PM_2.5_ exposure [52]. Similar results were obtained by the prospective cohort study by Lin Y.T. and colleagues [53], in that patients with CKD who were exposed to higher PM_2.5_ levels (> 32.08 µg/m³) had a significantly increased risk of progression of CKD into kidney failure, requiring replacement therapy (e.g., dialysis) (aHR = 1.42, 95% CI 1.12 to 1.80; p < 0.001). Moreover, an apparent dose-effect relationship was observed for a 7.8 µg/m³ IQR increment in the average 1-year PM_2.5_ exposure, which was significantly associated with a 19% greater risk of CKD progression (95% CI 1.08 to 1.31) [53]. However, no significant association could be shown between PM_2.5_ exposure and kidney failure requiring replacement therapy; furthermore, kidney failure requiring replacement therapy was significantly mediated by variability in 1-year estimated eGFR [53]. This suggests that nephrotoxic effects of PM_2.5_ might play a predominant role in CKD progression [51, 53].

All conducted studies evaluating the association between PM_2.5_ exposure and renal failure showed associations; not only development of CKD, ESRD, and renal failure have shown to be associated with PM_2.5_ exposure, but PM_2.5_ may also be involved in the progression from CKD to ESRD and eventually, renal failure.

### Kidney Transplantation

Vulnerable subpopulations, such as kidney transplant recipients, experience enhanced susceptibility due to triggering of the immune system by PM_2.5_, leading to inflammation [16, 56]. A retrospective cohort study by Pierotti et al. [56] found significant associations between the risk of kidney transplant failure and PM_2.5_ exposure. An increment of 5 µg/m³ PM_2.5_ was associated with an increased transplant failure risk (HR = 1.25, 95% CI 1.02 to 1.53); however, this association lost its significance after adjustment for confounders. The study concluded that there are no adverse effects of PM_2.5_ exposure on kidney transplant outcomes. However, a retrospective cohort study by Chang et al. [57] showed that higher baseline PM_2.5_ levels (the annual mean in the year before kidney transplantation), compared to the Q1 PM_2.5_ levels (1.2 to <8.3 µg/m³), were not associated with higher odds (aOR = 0.99, 95% CI 0.92 to 1.06) of acute kidney rejection for Q2 PM_2.5_ levels (8.3 to <9.8 µg/m³), but could be associated with increased odds (aOR = 1.11, 95% CI 1.04 to 1.20) for Q3 PM_2.5_ levels (9.8 to <11.9 µg/m³; p < 0.001). Feng Y. and colleagues [59] demonstrated that with each 10 µg/m³ increase in PM_2.5_ concentration, a 1.31-fold higher odds (95% CI 1.17 to 1.46) of one-year acute rejection was observed. That association was not present when the analysis was restricted to kidney transplant recipients who were exposed to PM_2.5_ concentrations ≤12 µg/m³ (OR = 1.02, 95% CI 0.87 to 1.19). Furthermore, Feng Y. et al. [59] stipulated that each 10 µg/m³ increase in PM_2.5_ was associated with a 1.59-fold (95% CI 1.46 to 1.73) higher odds of delayed graft function after transplantation; this association remained consistent, even when the analysis was restricted to kidney transplant recipients who were exposed to ≤12 µg/m³ PM_2.5_ (OR = 1.75, 95% CI 1.55 to 1.98).

Additionally, an increased risk of death-censored graft failure (aHR = 1.17, 95% CI 1.09 to 1.25) and all-cause mortality (aHR = 1.21, 95% CI 1.14 to 1.28) was shown per 10 µg/m³ increase in PM_2.5_ exposure levels. However, Feng Y. et al. [59] could not show an association between each 10 µg/m³ increase in PM_2.5_ and death-censored graft failure (HR = 1.05, 95% CI 0.97 to 1.15), but could associate all-cause mortality (HR = 1.15, 95% CI 1.07 – 1.23; p < 0.05). In a retrospective cohort study, Dehom et al. [58] also showed that a 10 µg/m³ increase in PM_2.5_ exposure levels granted an increased risk of all-cause mortality (HR = 3.45, 95% CI 3.08 to 3.78; p < 0.05) in kidney transplant recipients. Furthermore, results indicated that black recipients had a higher risk of all-cause mortality (HR = 4.09, 95% CI 3.43 to 4.88) than non-black recipients.

In short, results indicating an association between PM_2.5_ exposure and kidney transplant outcome are ambivalent. However, due to the scarce evidence about the mediating effects of pre-existing reduced kidney function on PM_2.5_ exposure and kidney function outcomes, it is too early to infer on the impact of PM_2.5_ exposure on kidney transplant survival. Further research is required. The occurrence of a pre-existing reduced kidney function and chronic immunosuppression might exacerbate the effects that PM_2.5_ exposure exerts on the kidneys and their functioning [56, 59].

### Limitations of Included Studies

The studies included in this systematic review hold some general limitations that must be addressed. Outdoor air pollution, such as PM_2.5_, has been investigated in association with e.g., atherosclerosis, hypertension, and coronary calcification. These conditions are mediators to kidney disease outcomes, which may cause indirect systemic detrimental effects on the kidney. However, although some studies in this review adjusted for these mediators in their statistical models, the effects exerted by PM_2.5_ on these mediators may aggravate and/or accelerate the kidney outcome investigated. Therefore, it is an interesting scope to investigate the mediation of e.g., hypertension in the PM_2.5_-kidney association, as has been done by Bowe et al. [34] for diabetes. Another limitation of the included studies is the homogeneity as to the examination of ethnic (sub)populations. Additional research is required on various polluted areas and ethnic (sub)populations to thoroughly investigate the impact of PM_2.5_ exposure on people’s renal health [40] and to extrapolate the findings to the general population as suggested by different authors [15, 23, 28, 29, 33–35, 37, 38, 40, 52, 56]. Furthermore, a substantial limitation is the absence of personalized PM_2.5_ exposure measurements or relying on only residential (regional or national) exposure levels. People do not spend their entire time during the day at the home address; therefore, all locations and the time spent at each location should be considered when determining exposure estimates [22, 30, 31, 37, 39, 53]. Even though PM_2.5_ has a greater specific surface area to facilitate the binding of toxic compounds, the composition of PM_2.5_ might heavily influence the adverse effects seen following exposure and might explain the vast differences seen between studies [91]. Residual confounding, such as smoking status, may be a limitation for determining the effects of PM_2.5_ exposure on an individual basis. However, when the studies treat the populations as one group, such as a time-series study, the influence of individual factors is minor. Moreover, the studies addressing the development and/or worsening of CKD did not always include comorbidity, (e.g., hypertension [36–38, 40]), and the stage of disease present at time of the inclusion [22, 36, 37, 39, 40, 51]. Furthermore, no studies investigated the underlying effects and mechanisms of PM_2.5_ exposure on specific kidney morphology and function or particulate biodistribution within the kidney. It would be interesting to investigate which structural renal components PM_2.5_ particles reach and potentially adversely affect.

## Conclusions and Future Directions

Epidemiological research assessed within this review revealed that PM_2.5_ air pollution presents significant public health risks, even at exposure levels below the previous standards set by the WHO [60]. Ran et al. [40, 52] stipulated that experimentally designed studies about the direct impact of PM_2.5_ on the renal system are still very limited [39, 51]. Causal evidence of the harmful effects of PM_2.5_ exposure on kidney function is still scarce, and the biological mechanisms of toxic action by which PM_2.5_ affects the kidneys or exacerbates kidney disease outcomes is not entirely elucidated until this day [34].

PM_2.5_ is an important, yet not fully recognized risk factor for kidney functioning and kidney disease outcome(s). On the other hand, because of the great variety of the investigated subpopulations, the contradictory findings, and the lack of sufficient studies addressing each subgroup of kidney disease(s), no summarizing consensus view can be reached across studies dealt within this systematic review. We conclude that more clarifying research is warranted to further elucidate the complex findings of PM_2.5_-linked effects on kidney function and kidney disease(s) to extrapolate the results to the general population and to evaluate the geographical variations in kidney disease(s) in the light of varying PM_2.5_ exposure levels.

## Supplementary Information

**Additional file 1: Supplementary Table 1.** Risk of bias analysis performed according to the Newcastle-Ottawa scale. Crosses (×) indicate the allocation of a point in the scale; The minus sign (-) indicates the failure to accommodate the subject(s) discussed. Ascertainment of exposure checks the derivation of the exposure measurements (×) and the demonstration that the outcome of interest was not present at the start of the study (×). Comparability encompasses the presence of the main confounder age (×) and any additional confounder(s) (×) respectively. Articles were considered to have a high risk of bias below 4 points, a medium risk of bias below 6 points, and a low risk of bias above or equal to 6 points.

## Data Availability

Data sharing is not applicable to this article as no datasets were generated or analysed during this systematic review.

## References

[1] Xu X, Nie S, Ding H, Hou FF (2018). Environmental pollution and kidney diseases. Nat Rev Nephrol.

[2] Arnold R, Issar T, Krishnan AV, Pussell BA. Neurological complications in chronic kidney disease. JRSM Cardiovasc Disease. 2016;5:2048004016677687. 10.1177/2048004016677687 PMC5102165 27867500

[3] Wang XH, Mitch WE (2014). Mechanisms of muscle wasting in chronic kidney disease. Nat Rev Nephrol.

[4] Bowe B, Xie Y, Li T, Yan Y, Xian H, Al-Aly Z (2019). Estimates of the 2016 global burden of kidney disease attributable to ambient fine particulate matter air pollution. BMJ Open.

[5] Lv J-C, Zhang L-X. Prevalence and disease burden of chronic kidney disease. Renal Fibrosis: Mechanisms and Therapies. 2019:3–15. 10.1007/978-981-13-8871-2_1 31399958

[6] System USRD. 2020 USRDS Annual Data Report: Epidemiology of kidney disease in the United States. National Institutes of Health, National Institute of Diabetes and Digestive and Kidney Diseases, Bethesda, MD, 2020.

[7] Loomis D, Grosse Y, Lauby-Secretan B, El Ghissassi F, Bouvard V, Benbrahim-Tallaa L, et al. The carcinogenicity of outdoor air pollution. Lancet Oncol. 2013;14(13):1262. 10.1016/s1470-2045(13)70487-x 25035875

[8] Elliott CT, Copes R. Burden of mortality due to ambient fine particulate air pollution (PM 2.5) in interior and Northern BC. Can J Public Health. 2011;102(5):390–3. 10.1007/BF03404182 PMC6973564 22032107

[9] Shaddick G, Thomas M, Mudu P, Ruggeri G, Gumy S (2020). Half the world’s population are exposed to increasing air pollution. NPJ Climate Atmospheric Science.

[10] Organization WH. WHO global air quality guidelines: particulate matter (PM2. 5 and PM10), ozone, nitrogen dioxide, sulfur dioxide and carbon monoxide: executive summary. 2021. 34662007

[11] Nemmar A, Hoet PM, Vanquickenborne B, Dinsdale D, Thomeer M, Hoylaerts M (2002). Passage of inhaled particles into the blood circulation in humans. Circulation.

[12] Bové H, Bongaerts E, Slenders E, Bijnens EM, Saenen ND, Gyselaers W, et al. Ambient black carbon particles reach the fetal side of human placenta. Nature Communications. 2019;10(1):1–7. 10.1038/s41467-019-11654-3 PMC6748955 31530803

[13] Maher BA, Ahmed IA, Karloukovski V, MacLaren DA, Foulds PG, Allsop D, et al. Magnetite pollution nanoparticles in the human brain. Proc Natl Acad Sci. 2016;113(39):10797-801. 10.1073/pnas.1605941113 PMC5047173 27601646

[14] Saenen ND, Bové H, Steuwe C, Roeffaers MB, Provost EB, Lefebvre W (2017). Children’s urinary environmental carbon load. A novel marker reflecting residential ambient air pollution exposure?. Am J Respir Crit Care Med.

[15] Zhao Y, Cai J, Zhu X, van Donkelaar A, Martin RV, Hua J (2020). Fine particulate matter exposure and renal function: A population-based study among pregnant women in China. Environ Int.

[16] Finn WF. Renal response to environmental toxics. Environ Health perspectives. 1977;20:15–26. 10.1289/ehp.772015 PMC1637353 598348

[17] Moher D, Weeks L, Ocampo M, Seely D, Sampson M, Altman DG (2011). Describing reporting guidelines for health research: a systematic review. J Clin Epidemiol.

[18] Becherucci F, Roperto RM, Materassi M, Romagnani P. Chronic kidney disease in children. Clin Kidney J. 2016;9(4):583–91. 10.1093/ckj/sfw047 PMC4957724 27478602

[19] Čukuranović R, Vlajković S. Age related anatomical and functional characteristics of human kidney. Organ. 2005;7:14.

[20] Wells G, Shea B, O’connell D, Peterson J, Welch V, Losos M, et al. The Newcastle-Ottawa Scale (NOS) for Assessing the Quality of Nonrandomized Studies in Meta-Analyses. Ottawa Health Research Institute: Ottawa, Ontario, 2010. 2015.

[21] Fang J, Tang S, Zhou J, Zhou J, Cui L, Kong F, et al. Associations between Personal PM2. 5 Elemental Constituents and Decline of Kidney Function in Older Individuals: the China BAPE Study. Environ Sci Technol. 2020;54(20):13167–74. 10.1021/acs.est.0c04051 32929958

[22] Blum MF, Surapaneni A, Stewart JD, Liao D, Yanosky JD, Whitsel EA (2020). Particulate Matter and Albuminuria, Glomerular Filtration Rate, and Incident CKD. Clin J Am Soc Nephrol.

[23] Mehta AJ, Zanobetti A, Bind MA, Kloog I, Koutrakis P, Sparrow D (2016). Long-Term Exposure to Ambient Fine Particulate Matter and Renal Function in Older Men: The Veterans Administration Normative Aging Study. Environ Health Perspect.

[24] Li A, Mei Y, Zhao M, Xu J, Li R, Zhao J (2021). Associations between air pollutant exposure and renal function: A prospective study of older adults without chronic kidney disease. Environ Pollut.

[25] Feng Y, Thijs L, Zhang Z-Y, Bijnens EM, Yang W-Y, Wei F-F, et al. Glomerular Function in Relation to Fine Airborne Particulate Matter in a Representative Population Sample. 2021. 10.1038/s41598-021-94136-1 PMC8290004 34282189

[26] Li Q, Wang Y-Y, Guo Y, Zhou H, Wang Q-M, Shen H-P (2021). Association between airborne particulate matter and renal function: An analysis of 2.5 million young adults. Environ Int.

[27] Gao X, Koutrakis P, Coull B, Lin X, Vokonas P, Schwartz J (2021). Short-term exposure to PM2. 5 components and renal health: Findings from the Veterans Affairs Normative Aging Study. J Hazard Mater.

[28] Chuang KJ, Pan CH, Su CL, Lai CH, Lin WY, Ma CM (2015). Urinary neutrophil gelatinase-associated lipocalin is associated with heavy metal exposure in welding workers. Sci Rep.

[29] Weaver AM, Wang Y, Wellenius GA, Young B, Boyle LD, Hickson DA (2019). Long-term exposure to ambient air pollution and renal function in African Americans: the Jackson Heart Study. J Expo Sci Environ Epidemiol.

[30] Xu X, Wang G, Chen N, Lu T, Nie S, Xu G (2016). Long-Term Exposure to Air Pollution and Increased Risk of Membranous Nephropathy in China. J Am Soc Nephrol.

[31] Lin S-Y, Hsu W-H, Lin C-L, Lin C-C, Lin C-H, Wang I, et al. Association of exposure to fine-particulate air pollution and acidic gases with incidence of nephrotic syndrome. Int J Environ Res Public Health. 2018;15(12):2860. 10.3390/ijerph15122860 PMC6313436 30558173

[32] Gu J, Shi Y, Zhu Y, Chen N, Wang H, Zhang Z (2020). Ambient air pollution and cause-specific risk of hospital admission in China: A nationwide time-series study. PLoS Med.

[33] Bernatsky S, Fournier M, Pineau CA, Clarke AE, Vinet E, Smargiassi A (2011). Associations between ambient fine particulate levels and disease activity in patients with systemic lupus erythematosus (SLE). Environ Health Perspect.

[34] Bowe B, Xie Y, Yan Y, Xian H, Al-Aly Z (2020). Diabetes Minimally Mediated the Association Between PM(2.5) Air Pollution and Kidney Outcomes. Sci Rep.

[35] Chin WS, Chang YK, Huang LF, Tsui HC, Hsu CC, Guo YL (2018). Effects of long-term exposure to CO and PM(2.5) on microalbuminuria in type 2 diabetes. Int J Hyg Environ Health.

[36] Bragg-Gresham J, Morgenstern H, McClellan W, Saydah S, Pavkov M, Williams D, et al. County-level air quality and the prevalence of diagnosed chronic kidney disease in the US Medicare population. PLoS ONE. 2018;13(7). 10.1371/journal.pone.0200612 PMC6067706 30063741

[37] Chan TC, Zhang Z, Lin BC, Lin C, Deng HB, Chuang YC, et al. Long-term exposure to ambient fine particulate matter and chronic kidney disease: A cohort study. Environ Health Perspectives. 2018;126(10). 10.1289/EHP3304 PMC6371647 30392394

[38] Chen SY, Chu DC, Lee JH, Yang YR, Chan CC (2018). Traffic-related air pollution associated with chronic kidney disease among elderly residents in Taipei City. Environ Pollut.

[39] Lin SY, Ju SW, Lin CL, Hsu WH, Lin CC, Ting IW (2020). Air pollutants and subsequent risk of chronic kidney disease and end-stage renal disease: A population-based cohort study. Environ Pollut.

[40] Ran J, Sun S, Han L, Zhao S, Chen D, Guo F (2020). Fine particulate matter and cause-specific mortality in the Hong Kong elder patients with chronic kidney disease. Chemosphere.

[41] Wang W, Wu C, Mu Z, Gu Y, Zheng Y, Ren L (2020). Effect of ambient air pollution exposure on renal dysfunction among hospitalized patients in Shanghai, China. Public Health.

[42] Yang YR, Chen YM, Chen SY, Chan CC (2017). Associations between Long-Term Particulate Matter Exposure and Adult Renal Function in the Taipei Metropolis. Environ Health Perspect.

[43] Jung J, Park JY, Kim YC, Lee H, Kim E, Kim YS, et al. Effects of air pollution on mortality of patients with chronic kidney disease: A large observational cohort study. Sci Total Environment. 2021;786:147471. 10.1016/j.scitotenv.2021.147471 33971609

[44] Ghazi L, Drawz PE, Berman JD. The association between fine particulate matter (PM2. 5) and chronic kidney disease using electronic health record data in urban Minnesota. J Exposure Sci Environ Epidemiol. 2021:1–7. 10.1038/s41370-021-00351-3 PMC8202050 34127789

[45] Liang Z, Wang W, Wang Y, Ma L, Liang C, Li P (2021). Urbanization, ambient air pollution, and prevalence of chronic kidney disease: A nationwide cross-sectional study. Environ Int.

[46] Bo Y, Brook JR, Lin C, Chang L-y, Guo C, Zeng Y, et al. Reduced Ambient PM2. 5 Was Associated with a Decreased Risk of Chronic Kidney Disease: A Longitudinal Cohort Study. Environ Sci Technol. 2021;55(10):6876–83. 10.1021/acs.est.1c00552 33904723

[47] Zeng Y, Lin C, Guo C, Bo Y, Chang L-y, Lau AK (2021). Combined effects of chronic PM2. 5 exposure and habitual exercise on renal function and chronic kidney disease: A longitudinal cohort study. Int J Hyg Environ Health.

[48] Li G, Huang J, Wang J, Zhao M, Liu Y, Guo X (2021). Long-term exposure to ambient PM2. 5 and increased risk of CKD prevalence in China. J Am Soc Nephrol.

[49] Kuźma Ł, Małyszko J, Bachórzewska-Gajewska H, Kralisz P, Dobrzycki S (2021). Exposure to air pollution and renal function. Sci Rep.

[50] Wu C-D, Chern Y-R, Pan W-C, Lung S-CC, Yao T-C, Tsai H-J, et al. Effects of surrounding environment on incidence of end stage renal disease. Sci Total Environment. 2020:137915. 10.1016/j.scitotenv.2020.137915 32392675

[51] Bowe B, Xie Y, Li T, Yan Y, Xian H, Al-Aly Z (2018). Particulate matter air pollution and the risk of incident CKD and progression to ESRD. J Am Soc Nephrol.

[52] Ran J, Yang A, Sun S, Han L, Li J, Guo F, et al. Long-Term Exposure to Ambient Fine Particulate Matter and Mortality from Renal Failure: A Retrospective Cohort Study in Hong Kong. Am J Epidemiol. 2020. 10.1093/aje/kwz282 31907517

[53] Lin Y-T, Lo Y-C, Chiang H-Y, Jung C-R, Wang C-M, Chan T-C, et al. Particulate Air Pollution and Progression to Kidney Failure With Replacement Therapy: An Advanced CKD Registry–Based Cohort Study in Taiwan. Am J Kidney Dis; 2020. 10.1053/j.ajkd.2020.02.447 32482472

[54] Bi J, Barry V, Weil EJ, Chang HH, Ebelt S. Short-term exposure to fine particulate air pollution and emergency department visits for kidney diseases in the Atlanta metropolitan area. Environ Epidemiol. 2021;5(4). 10.1097/EE9.0000000000000164 PMC8367053 34414347

[55] Feng Y, Jones MR, Chu NM, Segev DL, McAdams-DeMarco M (2021). Ambient air pollution and mortality among older patients initiating maintenance dialysis. Am J Nephrol.

[56] Pierotti L, Schofield SJ, Collett D, Fecht D, De Hoogh K, Hansell AL, et al. Traffic-related air pollution and solid organ transplant failure in Great Britain: A retrospective cohort study. J Transport Health. 2018;10:124–31.

[57] Chang S-H, Merzkani M, Murad H, Wang M, Bowe B, Lentine KL, et al. Association of ambient fine particulate matter air pollution with kidney transplant outcomes. JAMA network Open. 2021;4(10):e2128190-e. 10.1001/jamanetworkopen.2021.28190 PMC8498852 34618038

[58] Dehom S, Knutsen S, Shavlik D, Bahjri K, Ali H, Pompe L (2019). Long-Term Exposure to Fine Particulate Matter (PM2. 5) and Cardiovascular Disease Mortality among Renal Transplant Recipients. OBM Transplantation.

[59] Feng Y, Jones MR, Ahn JB, Garonzik-Wang JM, Segev DL, McAdams‐DeMarco M. Ambient air pollution and posttransplant outcomes among kidney transplant recipients. Am J Transplant. 2021. 10.1111/ajt.16605 PMC8500923 33870639

[60] WHO’s global air-quality guidelines (2006). Lancet.

[61] Levey AS, Coresh J, Tighiouart H, Greene T, Inker LA. Measured and estimated glomerular filtration rate: current status and future directions. Nature Reviews Nephrol. 2019:1–14. 10.1038/s41581-019-0191-y 31527790

[62] Levey AS, Stevens LA, Schmid CH, Zhang Y, Castro AF, Feldman HI (2009). A new equation to estimate glomerular filtration rate. Ann Intern Med.

[63] Levey AS, Bosch JP, Lewis JB, Greene T, Rogers N, Roth D (1999). A more accurate method to estimate glomerular filtration rate from serum creatinine: a new prediction equation. Ann Intern Med.

[64] Cheung KL, Lafayette RA (2013). Renal physiology of pregnancy. Adv Chronic Kidney Dis.

[65] Masson I, Flamant M, Maillard N, Rule AD, Vrtovsnik F, Peraldi M-N (2013). MDRD versus CKD-EPI equation to estimate glomerular filtration rate in kidney transplant recipients. Transplantation.

[66] Chen Y-W, Chen H-H, Wang T-E, Chang C-W, Chang C-W, Wu C-J. Difference between CKD-EPI and MDRD equations in calculating glomerular filtration rate in patients with cirrhosis. World J Gastroenterol. 2011;17(40):4532. 10.3748/wjg.v17.i40.4532 PMC3218145 22110285

[67] Eknoyan G, Lameire N, Eckardt K, Kasiske B, Wheeler D, Levin A (2013). KDIGO 2012 clinical practice guideline for the evaluation and management of chronic kidney disease. Kidney Int.

[68] Bikbov B, Perico N, Abbate M, Remuzzi G. The Glomerulus: Mechanisms and Patterns of Injury. Comprehensive Toxicology: Third Edition2017. p. 189-206.

[69] Pennemans V, De Winter LM, Munters E, Nawrot TS, Van Kerkhove E, Rigo J-M (2011). The association between urinary kidney injury molecule 1 and urinary cadmium in elderly during long-term, low-dose cadmium exposure: a pilot study. Environ Health.

[70] Genovese F, Manresa AA, Leeming DJ, Karsdal MA, Boor P. The extracellular matrix in the kidney: a source of novel non-invasive biomarkers of kidney fibrosis? Fibrogenesis Tissue Repair. 2014;7(1):4. 10.1186/1755-1536-7-4 PMC3986639 24678881

[71] Lamb EJ, Path F, Price CP. 21 Kidney Function Tests—Creatinine, Urea, and Uric Acid. Tietz Fundamentals of Clinical Chemistry and Molecular Diagnostics-E-Book. 2014:364.

[72] Shlipak MG, Matsushita K, Ärnlöv J, Inker LA, Katz R, Polkinghorne KR (2013). Cystatin C versus creatinine in determining risk based on kidney function. N Engl J Med.

[73] Argyropoulos CP, Chen SS, Ng Y-H, Roumelioti M-E, Shaffi K, Singh PP, et al. Rediscovering beta-2 microglobulin as a biomarker across the spectrum of kidney diseases. Front Med. 2017;4:73. 10.3389/fmed.2017.00073 PMC5471312 28664159

[74] Penders J, Delanghe JR. Alpha 1-microglobulin: clinical laboratory aspects and applications. Clin Chimica Acta. 2004;346(2):107–18. 10.1016/j.cccn.2004.03.037 15256311

[75] Norden AG, Lapsley M, Unwin RJ (2014). Urine retinol-binding protein 4: a functional biomarker of the proximal renal tubule. Adv Clin Chem.

[76] Ronco P, Debiec H. Pathophysiological advances in membranous nephropathy: time for a shift in patient’s care. Lancet. 2015;385(9981):1983–92. 10.1016/S0140-6736(15)60731-0 26090644

[77] Bernatsky S, Smargiassi A, Johnson M, Kaplan GG, Barnabe C, Svenson L, et al. Fine particulate air pollution, nitrogen dioxide, and systemic autoimmune rheumatic disease in Calgary. Alberta Environ Res. 2015;140:474–8. 10.1016/j.envres.2015.05.007 PMC4492844 25988990

[78] Faustini A, Renzi M, Kirchmayer U, Balducci M, Davoli M, Forastiere F. Short-term exposure to air pollution might exacerbate autoimmune diseases. Environ Epidemiol. 2018;2(3):e025.

[79] Darrow LA, Klein M, Flanders WD, Mulholland JA, Tolbert PE, Strickland MJ (2014). Air pollution and acute respiratory infections among children 0–4 years of age: an 18-year time-series study. Am J Epidemiol.

[80] Zhao J, Li M, Wang Z, Chen J, Zhao J, Xu Y (2019). Role of PM 2.5 in the development and progression of COPD and its mechanisms. Respir Res.

[81] Long M-h, Zhang C, Xu D-q, Fu W-l, Gan X-d, Li F (2020). PM2. 5 aggravates diabetes via the systemically activated IL-6-mediated STAT3/SOCS3 pathway in rats’ liver. Environ Pollut.

[82] Heck TG, Fiorin PBG, Frizzo MN, Ludwig MS. Fine Particulate Matter (PM2. 5) Air Pollution and Type 2 Diabetes Mellitus (T2DM): When Experimental Data Explains Epidemiological Facts. Diabetes Complications. 2018:71.

[83] Pecoits-Filho R, Abensur H, Betônico CC, Machado AD, Parente EB, Queiroz M (2016). Interactions between kidney disease and diabetes: dangerous liaisons. Diabetol Metab Syndr.

[84] Xu X, Liu C, Xu Z, Tzan K, Zhong M, Wang A (2011). Long-term exposure to ambient fine particulate pollution induces insulin resistance and mitochondrial alteration in adipose tissue. Toxicol Sci.

[85] Zelnick LR, Weiss NS, Kestenbaum BR, Robinson-Cohen C, Heagerty PJ, Tuttle K (2017). Diabetes and CKD in the United States population, 2009–2014. Clin J Am Soc Nephrol.

[86] Go AS, Chertow GM, Fan D, McCulloch CE, Hsu C-y (2004). Chronic kidney disease and the risks of death, cardiovascular events, and hospitalization. N Engl J Med.

[87] Kundu S, Stone EA. Composition and sources of fine particulate matter across urban and rural sites in the Midwestern United States. Environ Sci Proc Impacts. 2014;16(6):1360–70. 10.1039/c3em00719g PMC4191923 24736797

[88] Zhang Z, Chang L-y, Lau AK, Chan T-C, Chieh Chuang Y, Chan J (2017). Satellite-based estimates of long-term exposure to fine particulate matter are associated with C-reactive protein in 30 034 Taiwanese adults. Int J Epidemiol.

[89] Pugh D, Gallacher PJ, Dhaun N (2019). Management of hypertension in chronic kidney disease. Drugs.

[90] Hodgkins KS, Schnaper HW. Tubulointerstitial injury and the progression of chronic kidney disease. Pediatric Nephrol. 2012;27(6):901–9. 10.1007/s00467-011-1992-9 PMC3337413 21947270

[91] Pandey P, Patel D, Khan A, Barman S, Murthy R, Kisku G. Temporal distribution of fine particulates (PM2. 5, PM10), potentially toxic metals, PAHs and Metal-bound carcinogenic risk in the population of Lucknow City, India. J Environ Sci Health Part A. 2013;48(7):730–45. 10.1080/10934529.2013.744613 23445416

