# Supplementary material for: Adverse Effects of fine particulate matter on human kidney functioning: a systematic review
Source: Environ Health. 2022 Feb 8;21:24. doi: 10.1186/s12940-021-00827-7 (PMC8822715; doi:10.1186/s12940-021-00827-7)
Supplement: Supplementary file 1 — Additional file 1: Supplementary Table 1. Risk of bias analysis performed according to the Newcastle-Ottawa scale. Crosses (×) indicate the allocation of a point in the scale; The minus sign (-) indicates the failure to accommodate the subject(s) discussed. Ascertainment of exposure checks the derivation of the exposure measurements (×) and the demonstration that the outcome of interest was not present at the start of the study (×). Comparability encompasses the presence of the main confounder age (×) and any additional confounder(s) (×) respectively. Articles were considered to have a high risk of bias below 4 points, a medium risk of bias below 6 points, and a low risk of bias above or equal to 6 points. [file 12940_2021_827_MOESM1_ESM.pdf]

Supplementary Table 1. Risk of bias analysis performed according to the Newcastle-Ottawa scale.

Study ID	Selection			Comparability	Outcome		Total (Max. 9 ×)
	Representativeness of exposure cohort (×)	Selection of non-exposed cohort (×)	Ascertainment of exposure (××)	(××)	Assessment of outcome (×)	Adequacy of follow up (××)	
**Bernatsky *et al* (2011)**	×	N/A	× -	× ×	×	× ×	7×
**Bi *et al* (2021)**	×	N/A	× -	× ×	×	× ×	7×
**Blum *et al* (2020)**	×	N/A	× ×	× ×	-	× ×	7×
**Bo *et al* (2021)**	×	N/A	× ×	× ×	×	× ×	8×
**Bowe *et al* (2018)**	×	N/A	× ×	× ×	×	× ×	8×
**Bowe *et al* (2020)**	×	N/A	× ×	× ×	×	× ×	8×
**Bragg-Gresham *et al* (2018)**	×	N/A	× ×	× ×	×	× ×	8×
**Chan *et al* (2018)**	×	N/A	× ×	× ×	×	× ×	8×

**Chang *et al* (2021)**	×	N/A	× -	× ×	×	× ×	7×
**Chen *et al* (2018)**	×	N/A	× ×	× ×	×	× ×	8×
**Chin *et al* (2018)**	×	N/A	× ×	× ×	×	× ×	8×
**Chuang *et al* (2015)**	-	N/A	× ×	× ×	×	- ×	6×
**Dehom *et al* (2021)**	×	N/A	× ×	× ×	×	× ×	8×
**Fang *et al* (2020)**	-	N/A	× -	× ×	×	- ×	5×
**Feng *et al* (2021a)**	×	N/A	× ×	× ×	×	× ×	8×
**Feng *et al* (2021b)**	×	N/A	× ×	× ×	×	× ×	8×
**Feng *et al* (2021c)**	×	N/A	× ×	× ×	×	× ×	8×
**Gao *et al* (2021)**	×	N/A	× ×	× ×	×	× ×	8×
**Ghazi *et al* (2021)**	×	N/A	× ×	× ×	×	× ×	8×
**Gu *et al* (2020)**	×	N/A	× ×	× ×	×	× ×	8×

**Jung *et al* (2021)**	×	N/A	× ×	× ×	×	× ×	8×
**Kuźma *et al* (2021)**	×	N/A	× ×	× ×	×	× ×	8×
**Li A. *et al* (2021**	×	N/A	× ×	× ×	×	× ×	8×
**Li G. *et al* (2021)**	×	N/A	× -	× ×	×	× ×	7×
**Li Q. *et al* (2021)**	×	N/A	× ×	× ×	×	× ×	8×
**Liang *et al* (2021)**	×	N/A	× ×	× ×	×	× ×	8×
**Lin *et al* (2018)**	×	N/A	× ×	× ×	×	× ×	8×
**Lin *et al* (2020a)**	×	N/A	× ×	× ×	×	× ×	8×
**Lin *et al* (2020b)**	×	N/A	× -	× ×	×	× ×	7×
**Mehta *et al* (2016)**	×	N/A	× ×	× ×	×	× ×	8×
**Pierotti *et al* (2018)**	-	N/A	× ×	× ×	×	× ×	7×
**Ran *et al* (2020a)**	×	N/A	× ×	× ×	×	× ×	8×
**Ran *et al* (2020b)**	×	N/A	× ×	× ×	×	× ×	8×

**Weaver *et al* (2018)**	×	N/A	× -	× ×	-	× ×	6×
**Wang *et al* (2020)**	×	N/A	× ×	× ×	×	- ×	7×
**Wu *et al* (2020)**	×	N/A	× ×	× ×	×	× ×	8×
**Xu *et al* (2016)**	×	N/A	× ×	× ×	×	- ×	7×
**Yang *et al* (2017)**	×	N/A	× ×	× ×	×	- ×	7×
**Zeng *et al* (2021)**	×	N/A	× -	× ×	×	× ×	7×
**Zhao *et al* (2020)**	×	N/A	× ×	× ×	×	× ×	8×

Crosses (×) indicate the allocation of a point in the scale; The minus sign (-) indicates the failure to accommodate the subject(s) discussed. Ascertainment of exposure checks the derivation of the exposure measurements (×) and the demonstration that the outcome of interest was not present at the start of the study (×). Comparability encompasses the presence of the main confounder age (×) and any additional confounder(s) (×) respectively. Articles were considered to have a high risk of bias below 4 points, a medium risk of bias below 6 points, and a low risk of bias above or equal to 6 points. **Abbreviations:** N/A, not applicable.
